# Antiproliferative Effects of the Natural Oxadiazine Nocuolin A Are Associated With Impairment of Mitochondrial Oxidative Phosphorylation

**DOI:** 10.3389/fonc.2019.00224

**Published:** 2019-04-03

**Authors:** Maria Lígia Sousa, Marco Preto, Vítor Vasconcelos, Stig Linder, Ralph Urbatzka

**Affiliations:** ^1^Faculty of Sciences of University of Porto, Porto, Portugal; ^2^Interdisciplinary Centre of Marine and Environmental Research, Porto, Portugal; ^3^Department of Oncology and Pathology, Cancer Centre Karolinska, Karolinska Institute, Stockholm, Sweden; ^4^Department of Medical and Health Sciences, Linköping University, Linköping, Sweden

**Keywords:** natural products, cyanobacteria, spheroids, colon cancer, anti-cancer drugs, mitochondria, autophagy

## Abstract

Natural products are interesting sources for drug discovery. The natural product oxadiazine Nocuolin A (NocA) was previously isolated from the cyanobacterial strain *Nodularia* sp. LEGE 06071 and here we examined its cytotoxic effects against different strains of the colon cancer cell line HCT116 and the immortalized epithelial cell line hTERT RPE-1. NocA was cytotoxic against colon cancer cells and immortalized cells under conditions of exponential growth but was only weakly active against non-proliferating immortalized cells. NocA induced apoptosis by mechanism(s) resistant to overexpression of BCL family members. Interestingly, NocA affected viability and induced apoptosis of HCT116 cells grown as multicellular spheroids. Analysis of transcriptome profiles did not match signatures to any known compounds in CMap but indicated stress responses and induction of cell starvation. Evidence for autophagy was observed, and a decrease in various mitochondrial respiration parameter within 1 h of treatment. These results are consistent with previous findings showing that nutritionally compromised cells in spheroids are sensitive to impairment of mitochondrial energy production due to limited metabolic plasticity. We conclude that the antiproliferative effects of NocA are associated with effects on mitochondrial oxidative phosphorylation.

## Introduction

Historically, natural products were the primary source of drugs for medical preparations (1, 2). Despite the gradually abandonment of natural products by big pharma companies since the era of antibiotics discovery (3), natural products have returned into the focus as one of the most interesting sources for drug discovery (2, 4). Cyanobacteria are versatile microorganisms, adapted to many different environments and, as such, developed the capacity of producing secondary metabolites to overcome exogenous challenges (5–7). Their potential to produce secondary metabolites is already recognized with interesting biotechnological applications as bioplastics (8) or pharmaceuticals (9), and with anticancer (10, 11), antifouling (12, 13), antibiotics (14), or antiprotozoal activities (15, 16). Our own group showed that pico-cyanobacteria produce bioactive compounds that target cancer cells via different modes of actions (17). Hierridin B induced cell death probably via inhibition of mitochondrial activity and mitochondrial pores (18), while cytotoxic activity of portoamides was associated with disturbance of energy metabolism, and alterations in mitochondrial structure, and function (19). In the marine clinical pipeline (http://marinepharmacology.midwestern.edu/clinPipeline.htm), many compounds derived from cyanobacteria are in the different phases of clinical trials, and one compound (brentuximab vedotin) is already in clinical use for the treatment of malignant lymphoma and Hodgkin's disease (11).

Monolayer cell cultures are commonly used as model to screen for cytotoxic activity of new extracts or isolated compounds in cancer cell lines; however those culture conditions do not represent well the complexity of a tumor environment, namely the tumor hypoxic core, the highly prolific outer layer and the quiescent inner core, nutrient deprivation, penetration impair of the compounds, and often the limitation of their effects *in vivo* (20). The use of multicellular spheroids (MTS) as physiologically relevant models is assumed to decrease the risk of *in vivo* failures regarding the phenotypic screening of new compounds and the assessment of their effects (21). MTS are geometrically well-defined, which allows the direct relation with structure and function, and to discriminate the morphological zones of the tumor (necrotic, quiescent, and proliferative) (20, 22). Another advantage of MTS is the possibility to understand how compounds may act on the quiescent cells of the inner core, similar to chemoresistant cells, which is one of the main reason of failure of cancer treatment (23).

Nocuolin A (NocA) was isolated from the cyanobacterial strains Nostoc, Nodularia, and Anabaena by Voráčová et al. (24) and also chemically characterized. This compound has an unusual structure, a natural azole ring with N-N-O linkage and was found to induce apoptotic effects in the HeLa cell line and cytotoxic effects on other cancer cell lines (24). Using a bioassay-guided fractionation procedure, NocA was re-isolated from the cyanobacterial strain Nodularia sp. LEGE 06071 based on its cytotoxic activities in cancer cell lines (own unpublished data). The aim of the present work was to analyse the biological activity of NocA on colon cancer cells, and to evaluate its molecular mechanism of action. The effects of NocA on this type of cancer cells were not previously evaluated. Besides of its high frequency to cause cancer death (25), this cell type offers the possibility to study its comparative cytotoxic activity on monolayer cultures and MTS. Next generation sequencing was used as an unbiased approach to generate hypotheses about altered cellular signaling pathways. In order to test those hypotheses, functional assays focused on effects on cell survival, apoptosis, cell cycle, autophagy, mitochondrial respiration, and ER stress. The cytotoxic activity of NocA and functional assays were compared between cells grown as monolayer culture or as MTS in order to get insights into its potential effects on solid tumors.

## Methods

### Cyanobacteria Culture

The cyanobacterial strain LEGE 06071 was grown from the LEGE CC culture (26) of CIIMAR, Porto, Portugal. Cyanobacteria were cultured under standard conditions using Z8 medium supplemented with 20 μg L^−1^ vitamin B_12_ (27). Cultures were maintained at 25°C, under a light: dark cycle of 14:10 h, provided by cool white fluorescent tubes 10 μmol m^−2^ s^−1^. Cyanobacteria were harvested by centrifugation following by freeze-drying biomass.

### Nocuolin A Isolation

The initial extract was obtained from 39.2 g of lyophilized biomass of *Nodularia* sp. LEGE 06071, using a warm mixture of CH_2_Cl_2_: MetOH (2:1) yielding 8 g crude organic extract. The first fractionation was performed with a Vacuum-Liquid Chromatography (VLC) with a stationary phase of Silica Gel 60 (0.015–0.040, Merk KGaA). The mobile phase was a mixture of increasing polarity from 10% of EtOAc in hexane to 100% EtOAc to 100% MeOH. The samples were dissolved in methanol in a concentration of 1 mg ml^−1^ and analyzed by LC/MS on a Thermo Scientific LTQ Orbitrap XL spectrometer with a gradient from 20% MeCN (aq) to 100% MeCN (aq) for 20 min followed by isocratic condition at 100% MeCN for 10 min with a flow rate of 1 mL min^−1^. Fractions having NocA presence on the mass-spectrum [(M–H+) 299 (m/z)]; (M+Na+) = 321 (m/z); (2M+H+) = 597 (m/z); (2M+Na+) = 618(m/z) and tR = 6.44 min) were pooled together following a flash chromatography with crescent polarity solvents. The mobile phase was a mixture of increasing polarity from 40% of EtOAc in Hexanes to 100% EtOAc to 50% MeOH. Monitoring of the composition of the collected fraction was carried out using Thin Layer Chromatography (TLC), Silica Gel 60 F254 (Merk) under UV light with 254 and 366 nm. The TLC plates were also stained using Phosphomolybdic Acid Staining (PMA) at ~40°C and samples were pooled according to their TLC profile. Nine samples were then prepared for verification by LC-MS analysis as described before. Finally, NocA was isolated with a Waters HPLC System composed by a 1,525 binary pump and a UV-VIS detector, using a Synergi Fusion-RP column (4 μ 250 × 10 mm, 80 A) and a gradient elution program of 60–100% MeCN in 20 min, followed by 35 min at the same eluent composition, before returning to 60% MeCN in 5 min (flow = 3 mL/min). The abundance of NocA was checked by LC-MS and its purity accessed with ^1^H Nuclear Magnetic Resonance (NMR), BRUKER AVANCE III 400 MHz, 9.4 Tesla spectrometer equipped with a 5 mm cryoprobe. The NMR spectra were processed with Mnova software v8.0 (Mestrelab Research, S.L.).

### Cell Culture

The human colon carcinoma cell line HCT116^wt^ and breast cancer cell line MCF7^wt^ were obtained from the American Type Culture Collection, Manassas, VA, USA. The normal epithelial cell line hTERT RPE-1 (28) was obtained from Clontech (Palo Alto, CA) and HCT116 with red nucleus (red mKate2 fluorescent protein) was previously established using IncuCyte® NucLight Red Cell Labeling and according to manufacturer's instructions. HCT116 mutated cell lines (overexpression of BCL2, BCL2XL, MCL1) (29), and HCT116^pTRAFNrf2/HIF^ were provided by Prof Elias Arnér from Karolinska Institute, Sweden. All cell lines were sub-cultivated and grown in supplemented medium as recommended by the providers. All HCT116 colon carcinoma cell lines were maintained in McCoy's 5A modified medium, MCF-7 was grown in Dulbecco's Modified Eagle Medium (DMEM) supplemented with Glutamax^TM^ (Gibco, Massachusetts, USA) and hTERT RPE-1 was grown in Dulbecco's Modified Eagle Medium: Nutrient Mixture F-12 (DMEM/F12) supplemented with Glutamax^TM^ (Gibco, Massachusetts, USA). All media were also supplemented with 10% fetal bovine serum (Biochrom, Berlin, Germany), 1% of penicillin/streptomycin (Biochrom, Berlin, Germany), and 0.1% of Amphotericin B (GE Healthcare, Little Chafont, United Kingdom), and all cells lines incubated at 37°C in 5% CO_2_.

### Generation of MTS

After confirming a pre-confluent growth (over 80%) on a monolayer culture, the cells were detached from the plates by trypsinisation and collected. Two hundred microliter of medium containing 50,000 cells are added on each well of a 96 well-plate (U-round bottom ultra-low attachment, Corning). Then 190 μl of medium was added, so that top of the well should form a drop, resulting of the excess of medium. Elevating the lid from the plate with plasticine on the corners, the plate was firmly putted upside down resulting in a drop by superficial tension on each well. The plate was then incubated over 24 h with shaking (300 rpm) under 37°C and humidified atmosphere with 5% of CO_2_. On the next day, the plate was flipped back to the normal position. The excess of medium (190 μl) was removed and then the newly-formed spheroids were incubated for 5 days.

### Glucose/Galactose Assay

For the Glucose/Galactose assay HCT116^wt^ cells were seeded at 3.3 × 10^4^ cells mL^−1^ in 96 well-plates and after 24 h they were changed to glucose (25 mM) or galactose (10 mM) conditioned media, respectively, as described previously by Eakins et al. (30) and exposed to NocA at concentrations up to 10 μM over 48 h. After that period, MTT assay was performed as described below.

### Viability Analyses

Cells were seeded at 3.3 × 10^4^ cells mL^−1^ in 96 well-plates and after 24 h exposed to NocA concentrations up to 10 μM and kept in the incubator for 48 h. After the exposure, 3-(4,5-dimethylthiazol-2-yl)-2,5-diphenyltetrazolium bromide) (MTT) was added at a final concentration of 0.05 mg ml^−1^ per well for 3 h. The formation of formazan crystals was visually evaluated by microscopy, dissolved in DMSO and absorbance measured at 550 nm on a GEN5TM-Multi-detection Microplate Reader (Biotek, Bad Friedrichshall, Germany). All compounds tested in this work were dissolved in dimethylsulphoxide (DMSO) (Sigma, USA). A maximum final concentration of 0.5% DMSO was reached in cell cultures; control wells received solvent only. Suforhodamide B colorimetric assays was also performed in HCT116 cell line cultured and exposed at the same conditions and the viability assay was performed according to Vichai and Kirtikara (31).

The size development of MTS was followed after exposure for 48 h in the IncuCyte-FLR 20X phase contrast/fluorescence microscope (Essen Instruments, Ann Arbor, MI). Images of spheroids were taken every 4 h, and spheroids area was measured every 24 h aided by Incucyte ZOOM Live-Cell Analysis System (Essen Instruments, Ann Arbor, MI).

### Incucyte Analysis of Nrf2 and HIF

For analysis of Nrf2 and HIF, a HCT116 cell line transformed with a pTRAF^Nrf2/HIF^ plasmid were cultured as described before. NocA was tested on a concentration-response assay (up to 10 μM) and incubated on an Incucyte ZOOM Live-Cell Analysis System (Essen Instruments, Ann Arbor, MI). For the duration of 48 h, Nrf2 activity was read at λex = 585 nm, emission filter: 635 nm, and auranofin (2 μM) was used as a positive control to confirm Nrf2 activity and HIF activity was read λex = 460 nm, emission filter: 524 nm using VLX600 (32) as positive control, at a concentration of 6 μM.

### Autophagy Staining

HCT116 cells were seeded on a top of sterile cover slide and let adhere for 24 h. Then, cells were exposed to IC_90_ concentration of NocA (7 μM) over 6 and 18 h. At the end of the exposure, cells were stained with 80 μM of monodansylcadaverine (MDC) for 10 min at 37°C in the dark. The slides were then mounted and visualized on a fluorescent microscope Leica DM6000B under DAPI filter and images were analyzed with Cell Profiler software https://cellprofiler.org/. MTS were also stained with MDC at 80 μM for 10 min at 37°C, 5% CO_2_ in the dark and then visualized under the same fluorescent microscope.

### Determination of Apoptosis by ELISA

Following drug exposure, caspase-cleaved keratin-18 (K18-Asp396) was determined following the instructions of the manufacturer using the M30-Cytodeath ELISA developed for detection of soluble caspase-cleaved keratin 18 (VLVBioPeviva AB, BrommaStockholm, Sweden).

### Western Blot

Cells were collected in RIPA buffer (150 mM NaCl, 50 mM Tris, pH 7.4, 1% Nonidet P-40, 0.1% SDS, 0.5% sodium deoxycholate) supplemented with protease and phosphatase inhibitors. The protein concentration of cellular extracts was determined by Pierce Coomassie Plus (Bradford) Assay Reagent. SDS-PAGE was performed on pre-casted acrylamide gels NuPAGE™ 4–12% Bis-Tris Protein (Invitrogen™) using the NuPage system and loading 20 μg of proteins per lane. After electrophoresis, proteins were transferred to nitrocellulose membranes and incubated for 1 h at room temperature in PBS-Tween with 5% non-fat dry milk. Protein loading was assessed by red Ponceau staining of the membranes. Primary antibodies were used according to the manufacturer instructions, LC3 (#2775) (1:1,000), Phospho-eIF2 α (Ser51) (#9721) (1:1,000), and eIF2 α (#9722) (1:1,000), all antibodies were from Cell Signaling Technology (Danvers, MA, USA). Antibody to β-actin (#A5316) was from Sigma-Aldrich (St Louis, MO, USA) (1:5,000). Membranes were incubated with primary antibodies dilutions in 5% w/v BSA, 1x TBS, 0.1% Tween-20 overnight at 4°C, followed by appropriate HRP-conjugated secondary antibodies. Secondary Anti-rabbit IgG HRP-linked (#7074) and Anti-mouse IgG, HRP-linked Antibody (#7076), (Cell Signaling Technology, Danvers, MA, USA) were diluted at 1:5,000 in 5% non-fat milk and incubated 1 h at room temperature. Antibody staining was visualized by the Clarity Western ECL Substrate (BioRad) on a BioRad Molecular Imager Gel Doc^TM^ XR+ with Image Lab^TM^ Software.

### ATP Determination

ATP levels were determined according to Mitochondrial ToxGlo™ Assay kit (Promega, Madison, WI). HCT116 cells were seeded at a density 2.0 × 10^5^ cells mL^−1^ in a 96-well plate, overnight incubation 37°C, 5% CO_2_ to allow the adhesion of the cells. Then, cellular ATP levels were determined relative to vehicle-treated control cells after 2 h exposure period to seven concentrations of NocA (up to 10 μM, with 1:2 dilutions). Cytotoxicity reagent from the kit was added and fluorescence measured at 485 nm_Ex_/520–530 nm_Em_. Following, ATP detection reagent was added, and luminescence measured on a Spark® multimode microplate reader (Tecan).

### Mitochondrial Respiration

HCT116 cells with a density of 6 × 10^4^ cells ml^−1^ of were seeded on a XF24-plate containing blank controls. The Seahorse XF analyzer was used as indicated by the manufacturer (Seahorse Bioscience). Before the measurements, medium was replaced with 500 μL of Seahorse XF base medium (200 mM L-glutamine, 1 mM pyruvate, 2.5 M glucose, pH 7.4) at 37°C without CO_2_ for 1 h. Oxygen consumption rate (OCR) values were measured by XF24 Extracellular Flux Analyzer. The assessment of Mitochondrial activity was performed using a Seahorse XF Cell Mito Stress Test Kit (Seahorse Bioscience). All experiments were performed in triplicates and were repeated three times.

### Flow Cytometry

For cell cycle analysis, HCT116 cells were seeded at a density of 5 × 10^5^ cells ml^−1^ in 24 well-plates and exposed to Paclitaxel 100 nM (33), NocA 2.5 μM, and NocA 1.25 μM for 24 and 48 h. After exposure, cells were collected, fixed in 70% ethanol and stored at −20°C. On the day of the analysis, the cells were collected again and resuspended in 100 μl of PI/RNAse staining buffer (BD Pharmingen™, USA) for 40 min. Then 400 μl of PBS was added and cells were analyzed on a BD FACSCanto™ II flow cytometer. Cells were gated on the basis of the forward (FSC) and side scatter (SSC) profiles and were analyzed with a BD FACSCanto II equipment (Bekton Dickinson) and 10,000 events per sample were collected. For apoptosis, cells were seeded as described for cell cycle analysis, but the cells were lived stained with 5 μl of FITC Annexin V (Immunotools, Germany) and 10 μl Propidium Iodide (PI) at 50 μg ml^−1^ freshly prepared in 100 μl PBS Ca^2+^ binding buffer for 20 min. Four hundred microliter of binding buffer was added before analysis on a BD Accuri 6 Flow Cytometer. Ten thousand events, gated based on FSC and SSC, were collected on FL1/FL3 channels. For mitochondrial depolarization analysis, HCT 116 cells were seeded at a density of 5 × 10^5^ cells ml^−1^ in 6 well-plates and exposed with 7 μM of NocA over 6 h. Cells were incubated with 25 nM TMRE (Invitrogen) for 30 min and changes in mitochondrial membrane potential (ΔΨ) were monitored in an Gallios Flow Cytometer, cells gated according to FSC And SSC profile, and 60,000 events were collected on FL2 channel per sample (34). Data analysis of flow cytometry data was performed using FlowJo v10 (FlowJo LCC, Ashland, OR, USA).

### RNA Extraction

MCF-7 cells were cultured as described by Lamb et al. (35). After exposure, cells were washed with PBS, and total RNA was prepared using RNeasy miniprep kit (Qiagen, Chatsworth, CA) with a removal of DNA contamination step by DNAse I digestion (BioRad Laboratories, USA). The quantification of RNA was determined on a Biotek, HT Synergy reader using a Take3™ multi-volume plate, and the quality of RNA was verified on an 1% agarose gel. Then, the samples were sent to GATC Biotech AG (Konstanz, Germany) for RNA sequencing. Single-end samples were aligned to the reference Human genome hg19.chronly/GRC37, UCSC with Gencode v19, and Ensembl 75 annotations. Of a total of 55,850,897 reads on control sample, 99% were mapped and from 62,514,401 on NocA 99.1% of NocA reads were mapped.

### Bioinformatic Analysis of RNAseq

As main NGS results, a list of genes (19,976) was obtained and its number of reads corrected for sequence length bias. For analysis of C-Map, the 150 most up-regulated genes (maximum absolute fold ranging 159.36 to 5.12) and 150 most down-regulated genes (absolute minimum fold ranging −3.51 to −208.33) were inserted. A query on C-Map (https://clue.io/l1000-query) (36) should retrieve a list of perturbagens that can be used to find similar compounds, gene knock-down, gene over-expression, and C-Map classes with similar expressing signatures to NocA. The C-Map classes with connectivity score (τ) higher than 90 were selected, as having significant relevance (36). Gene-set enrichment analysis (GSEA) was performed for the complete gene list using GSEA software (37) with 1,000 permutations on the gene set, and default values for other parameters. The gene set was selected from MSigDB C5 collection: Gene Ontology (GO) gene sets (http://www.broadinstitute.org/gsea/msigdb/collections.jsp). The enriched GO Pathways with nominal values (the statistical significance of the enrichment score) lower than 0.005 were exported to Cytoscape 3.6.0 and an enrichment analysis map was constructed (38, 39).

### Statistical Analysis

The IC_50_ values were calculated from dose-response curves of NocA after transforming the data to log scale and normalization, defining 0% of viability as the positive control and the 100% of viability the solvent control. A non-linear regression choosing a variable slope with four parameters was performed and IC_50_ calculated on the inflection point. Data was retrieved from three independent experiments with ≥3 replicates each. ATP levels obtained by the Mitochondrial ToxGlo™ Assay kit were transformed to a log scale and normalized defining 0% of viability as positive control and 100% viability as solvent control, and ATP minimum values 0% with the mito-toxicant CCCP at 10 μM, and untreated wells as 100% ATP values. Error bars of the graphs represent the standard deviation. Apoptosis analysis from flow cytometry assay, Seahorse results and morphological alterations of spheroids imaged on IncuCyte were analyzed with a two-way ANOVA followed by a Bonferroni Multi comparison *post hoc*-test. Flow Cytometry were performed in triplicates counting 10,000 events on each sample, whereas Seahorse was analyzed with *n* = 6 over three independent experiments, and IncuCyte with *n* = 6. Western blot statistical analysis was performed using protein bands signal of three independent experiments with a one-way ANOVA followed by Bonferroni Multicomparison *post-hoc*-test. Results from M30-Cytodeath ELISA and fluorescence staining of spheroids were analyzed with one-way ANOVA followed by Dunnett's Multiple Comparison Test, using three independent assays with each in triplicates. For all analyses, the confidence interval was 95%, where ^*^p < 0.05; ^**^p < 0.01; ^***^p < 0.001. Error bars of the graphs represent the standard deviations.

## Results

### NocA Shows Antiproliferative Effects on HCT116 Colon Cancer Cells in Monolayer and Spheroid Cultures

Nocuolin A (NocA) was isolated from the cyanobacterial strain LEGE 06071 and the purity estimated to ~99% by LC-MS (Supplementary Figure 1) and ^1^H-NMR analysis (Supplementary Figure 2). Under conditions of exponential cell proliferation, NocA had similar antiproliferative activities on HCT116 colon cancer cells and immortalized human hTERT-RPE-1 epithelial cells using the MTT assay (IC_50_ values of 1.35 ± 0.96 and 1.2 ± 0.6 μM, respectively). Since proliferating normal cells are sensitive to cancer therapeutic agents, we also examined the effects of NocA on hTERT-RPE-1 cells under non-proliferating conditions (Supplementary Figure 3B), using confluent and contact inhibited hTERT-RPE-1 cultures (40). When confluent cultures of hTERT-RPE-1 cells were exposed to NocA, an IC_50_ value of >10 μM was observed (Supplementary Figure 3A).

**Figure 1:**
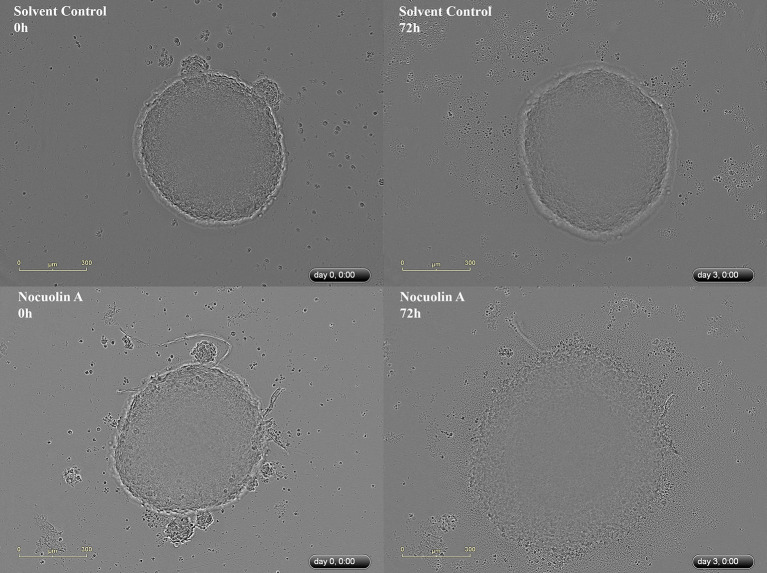
Representative IncuCyte images of HCT116^wt^ spheroids. Top image represents a spheroid in the beginning (0 h) and at the end of the experiment (72 h), without exposure to NocA while the bottom images represent a spheroid exposed to 20 μM of NocA. Quantitative analysis is shown on Supplementary Figure 4 derived from 3 independent experiments in triplicates.

The multicellular tumor spheroid (MTS) model is of intermediate complexity between *in vivo* tumors and monolayer cell cultures. The response of HCT116 MTS to different concentrations of NocA was evaluated using an IncuCyte® instrument. NocA showed a disaggregation of the outer cell layers in particular at concentrations >5 μM (Figure 1 and Supplementary Figure 4) leading to an increase of the spheroid size. Additionally, a loss of fluorescence signal of HCT116 red nucleus MTS indicated reduced viability in the outer layer of the spheroid (Supplementary Movies).

### NocA Induced Apoptosis of Colon Cancer Cells in Both Monolayer and Spheroid Cultures

**Figure 2:**
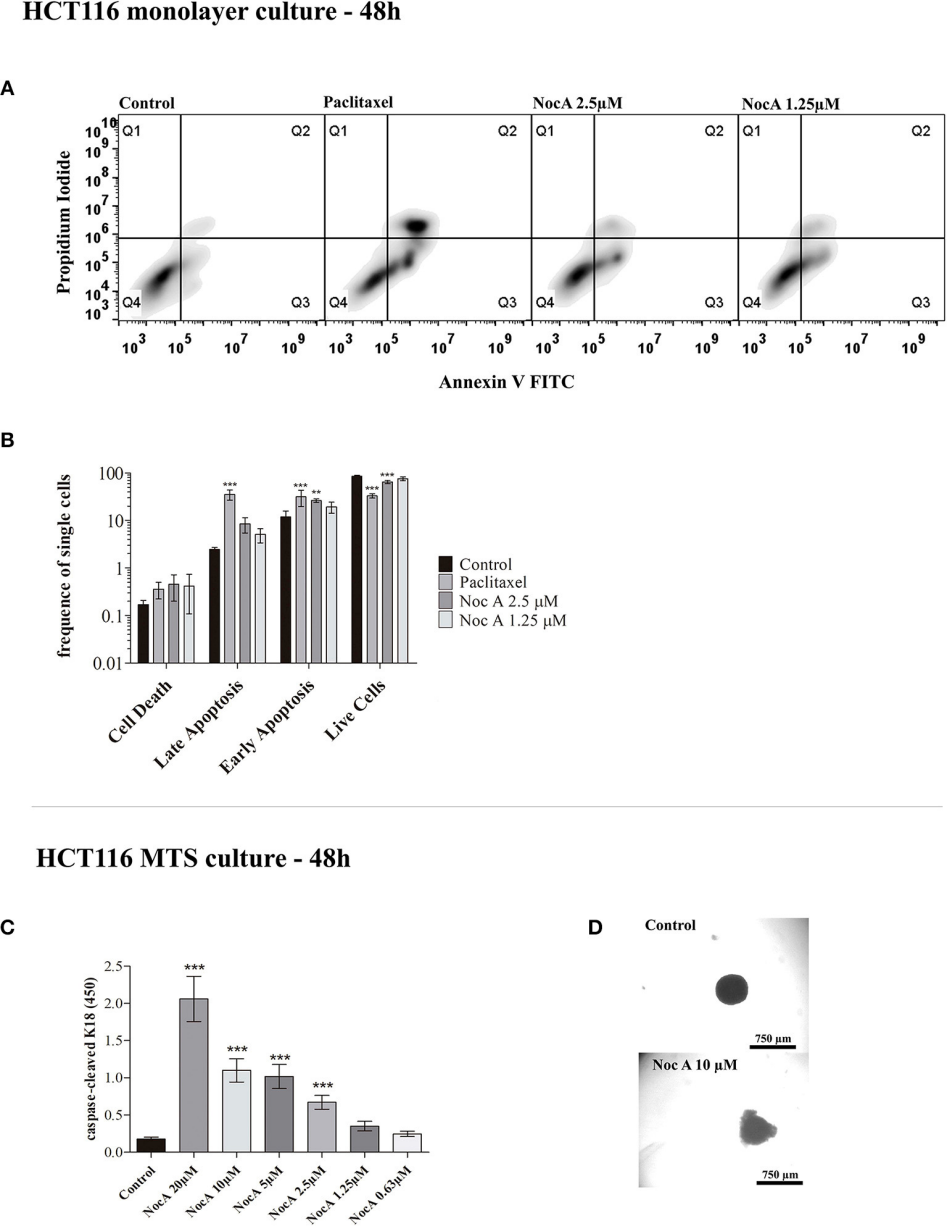
Analysis of apoptosis on monolayer culture and multicellular spheroids (MTS). **(A)** Apoptosis analysis of monolayer culture HCT116 cells using flow cytometry with Annexin V and Propidium iodide staining. **(B)** Quantifications of flow cytometry results. Apoptosis is observed after 48 h at 2.5 μM. ^***^p < 0.001; ^**^p < 0.01. Ten thousand cells per gated replicate were counted, *n* = 3. **(C)** M30 CytoDeathTM ELISA absorbance readings on HCT116 MTS exposed to NocA up to 20 μM. High absorbance values represent higher levels of caspase-cleaved K18. Two independent assays were performed, *n* = 3. **(D)** Bright field images of MTS after 48 h of exposure, being the top image a control and the bottom image an MTS exposed to Noc A at 10 μM; ^***^p < 0.001.

To examine the mechanism of antiproliferative effects, we first analyzed whether the cell cycle was affected using propidium iodide staining and flow cytometry. No effect on the cell cycle was evident after 24 or 48 h of exposure to NocA (Supplementary Figure 5). We next investigated possible induction of apoptosis using annexin V/propidium iodide staining and flow cytometry (Figure 2A). This analysis demonstrated that apoptosis occurred in cells exposed to NocA for 48 h. Apoptosis was observed at a concentration close to the IC_50_ suggesting that apoptosis was the underlying mechanism for antiproliferative effects. Significant apoptosis was not observed after 24 h exposure (Figure 2B).

BCL2 family proteins are instrumental to apoptosis involving the mitochondrial pathway (29, 41). HCT116 cells overexpressing the genes *BCL2, MCL1*, or *BCL2XL* proteins were exposed to increasing concentrations of NocA for 48 h. However, cell viability was not significantly affected by overexpression of these proteins (Table 1). We next examined whether NocA would induce apoptosis in HCT116 MTS (Figure 2D). An endogenous caspase-cleavage product (CK18-DALD_396_, ccCK18) was assessed using the M30-Apoptosense® enzyme-linked immunosorbent assay method (21). Increased levels of ccCK18 were observed at concentrations ≥2.5 μM (Figure 2C). Drugs that induce apoptosis of tumor cells, commonly induce an oxidative stress response (42). We examined the response to NocA using a HCT116 reporter cell line where antioxidant/electrophile response element (ARE/EpRE) are under the control of the Nrf2 promoter (43). No effect of NocA was observed over 48 h of exposure (Supplementary Figure 6), which indicated no induction of oxidative stress.

**Table 1:** IC_50_ for NocA cytotoxic activity in colon cancer cell line HCT116^wt^ and HCT116 mutated cell lines, BCL family proteins overexpression, and MCF7^wt^ cell line.

**Cell line**	**IC_**50**_ [μM] mean ± SD**
HCT116^wt^	1.3 ± 0.9
HCT116^BCL2+/+^	1.2 ± 0.4
HCT116^BCL2XL+/+^	1.6 ± 0.6
HCT116^MCL1+/+^	1.9 ± 0.6
MCF7^wt^	1.6 ± 0.6

*Viability was analyzed by MTT assay after 48 h of exposure with n > 4 in three independent assays*.

### Analysis of Transcriptome Profiles Shows a Complex Response

To generate hypotheses regarding the mechanism of action of NocA, we examined transcriptome signatures from drug-treated cells (35). The Connectivity Map database (Broad Institute) is based mainly on the MCF7 breast cancer cell line and we first established that NocA shows antiproliferative activities in this cell line (IC_50_ of 1.6 μM ± 0.6) (Supplementary Figure 3C). The transcriptome profile was determined after 6 h exposure of MCF7 cells to 7 μM NocA and signatures with connectivity score (τ) higher than 90 were selected (35, 36, 44). These gene set signatures were rank-ordered by similarity of differentially-expressed gene sets of MCF7 and, in addition, by similarity of a summary connectivity score across nine cell lines (A375, A549, HCC515, HEPG2, HT29, MCF7, PC3, HA1E, VCAP) (Table 2). The query comparing connectivity with the summary across the nine cell lines on the database demonstrated five CMap pharmacological classes with strong connectivity score, similar to the classes obtained for MCF cell line analysis (IKK inhibitor, protein synthesis inhibitor, ATPase inhibitor, PKC activator, T-type calcium channel blocker). The exceptions were T-type calcium channel blocker, which only appeared with connectivity for MCF7 cell analysis, and BCL inhibitor, which only had strong connectivity on the summary across all the cell lines (Table 3).

**Table 2:** Detailed list of connections of transcriptome of MCF7 cells exposed to NocA to reference perturbagens of C-Map MCF7 cell line.

**Rank**	**Score (τ)**	**Name**	**Compounds**	**Target**
**MCF7**				
10.00	98.94	Vesicular Transport		COPA; COPB2
22.00	98.37	IKK inhibitor	Withaferin-a; IKK-16; IKK-2-inhibitor-V; BX-795; TPCA-1	
35.00	97.50	Protein synthesis inhibitor	Brefeldin-a; puromycin; homoharringtonine; emetine; cycloheximide; cephaeline	
44.00	96.89	ATPase inhibitor	Strophanthidin; digitoxin; cyclopiazonic-acid; digoxin; cinobufagin; cymarin; bufalin; ouabain; proscillaridin; digitoxigenin	
133.00	90.74	PKC activator	Prostratin; phorbol-12-myristate-13-acetate	
137.00	90.59	T-type calcium channel blocker	Penfluridol; dichlorobenzamil; KB-R7943	

*Score (τ) stands for the fraction of reference gene sets with a greater similarity to the perturbagen*.

**Table 3:** Detailed list of connections of transcriptome of MCF7 cells exposed to NocA to reference perturbagens of C-Map summary across the cell lines available in the database (A375, A549, HCC515, HEPG2, HT29, MCF7, PC3, HA1E, VCAP).

**Rank**	**Score (τ)**	**Name**	**Compound**
**9 CELL LINES**			
1.00	99.02	Protein synthesis inhibitor	Emetine; cycloheximide; homoharringtonine; cephaeline; verrucarin-a; brefeldin-a; puromycin
4.00	98.13	ATPase inhibitor	Cinobufagin;bufalin; ouabain; proscillaridin; digoxin; digitoxin; strophanthidin; cymarin; cyclopiazonic-acid; digitoxigenin
12.00	97.47	PKC activator	Prostratin; ingenol; phorbol-12-myristate-13-acetate
20.00	96.58	IKK inhibitor	BX-795; IKK-2-inhibitor-V
28.00	95.20	BCL inhibitor	ABT-737; obatoclax

*Score (τ) stands for the fraction of reference gene sets with a greater similarity to the perturbagen*.

**Figure 3:**
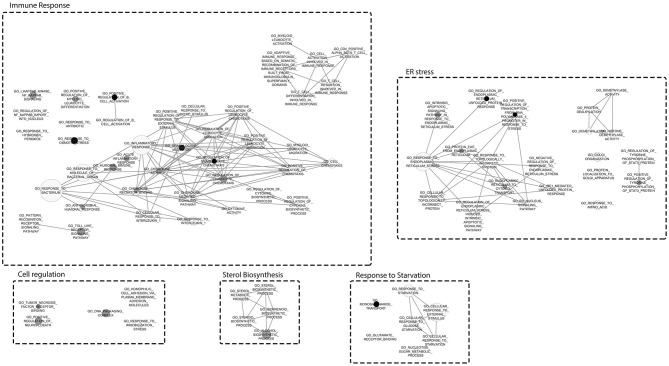
Gene Set Enrichment Analysis mapped clustered with Cytoscape. Clusters represent different responses of exposed cells to NocA. Node sizes represent the number of genes and darken of the color increases with significance (FDR *Q*-value Cut-off = 0.1; *P*-value Cut-off = 0.005).

A Gene Set Enrichment Analysis by using the transcriptome data demonstrated the enrichment of 79 GO terms for NocA treated cells compared to the solvent control (Supplementary Table 1). Major cluster of cellular dysregulations were identified and belonged to the immune response, stress response, sterol biosynthesis, response to starvation, and endoplasmic reticulum stress. According to the number of enriched genes and difference in read frequencies, the most enriched GO terms were organized in clusters to facilitate the visualization of the data by Cytoscape software (Figure 3). We conclude from these data that the response to NocA is complex and is characterized by an expected general stress response but also response to starvation and endoplasmic reticulum stress.

### Evidence of Autophagy Induction

**Figure 4:**
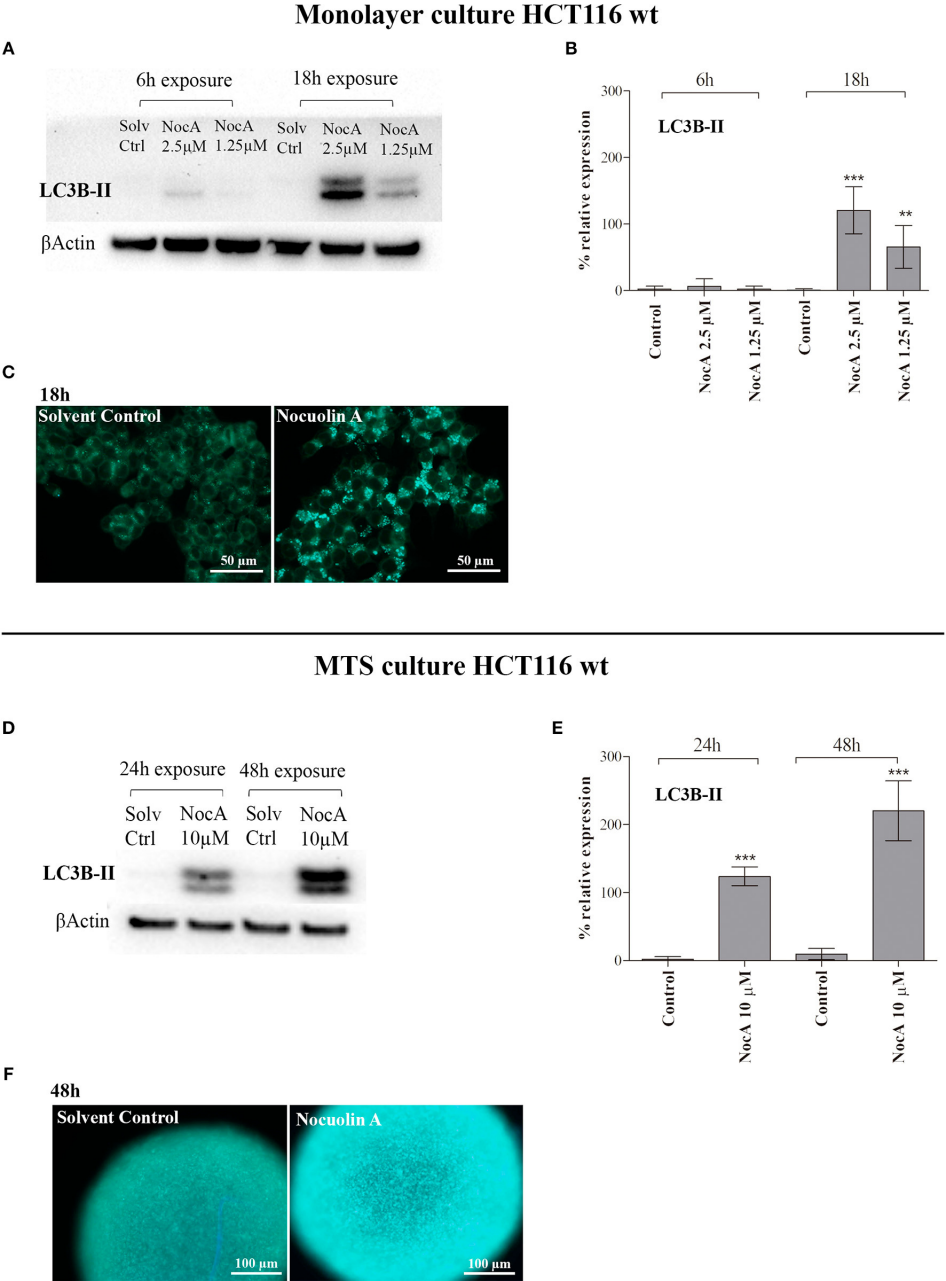
Evidence of autophagy induction on monolayer cell culture of HT116 and multicellular systems (MTS). **(A)** Western blot of HCT116 cells exposed to NocA at 2.5 and 1.25 μM for 6 and 18 h with detection of LC3-B **(B)**. **(C)** MDC staining of autophagic compartments on HCT116^wt^ monolayer cultured cells exposed to NocA 7 μM over 18 h. **(D)** Western blot of MTS of HCT116 cells exposed to NocA at 10 μM for 24 and 48 h with detection of LC3B-II. **(E)** LC3B-II quantification. **(F)** MDC staining of MTS exposed to NocA 7 μM over 48 h. Three independent assays, ^***^p < 0.001; ^**^p < 0.01.

Based on the finding of response to starvation in the GSEA analysis raised the possibility that NocA may induce autophagy. The LC3B-II protein expression of cells exposed to NocA at concentrations of 1.25–2.5 μM was significant increased after 18 h of exposure (Figures 4A,B). Induction of LC3B-II was also after observed 24 and 48 h of exposure of MTS to NocA (Figures 4D,E). Monodansylcadaverine (45) is a stain of autophagic compartments, and fluorescence intensity after 18 h of exposure to NocA was increased in HCT116 cells in monolayer cultures and on MTS after 48 h of exposure (Figures 4C,F). These results, although preliminary, led to the hypothesis that NocA has a negative effect on cell metabolism.

### NocA Induced a Decrease in Cellular ATP and Mitochondrial Respiration

**Figure 5:**
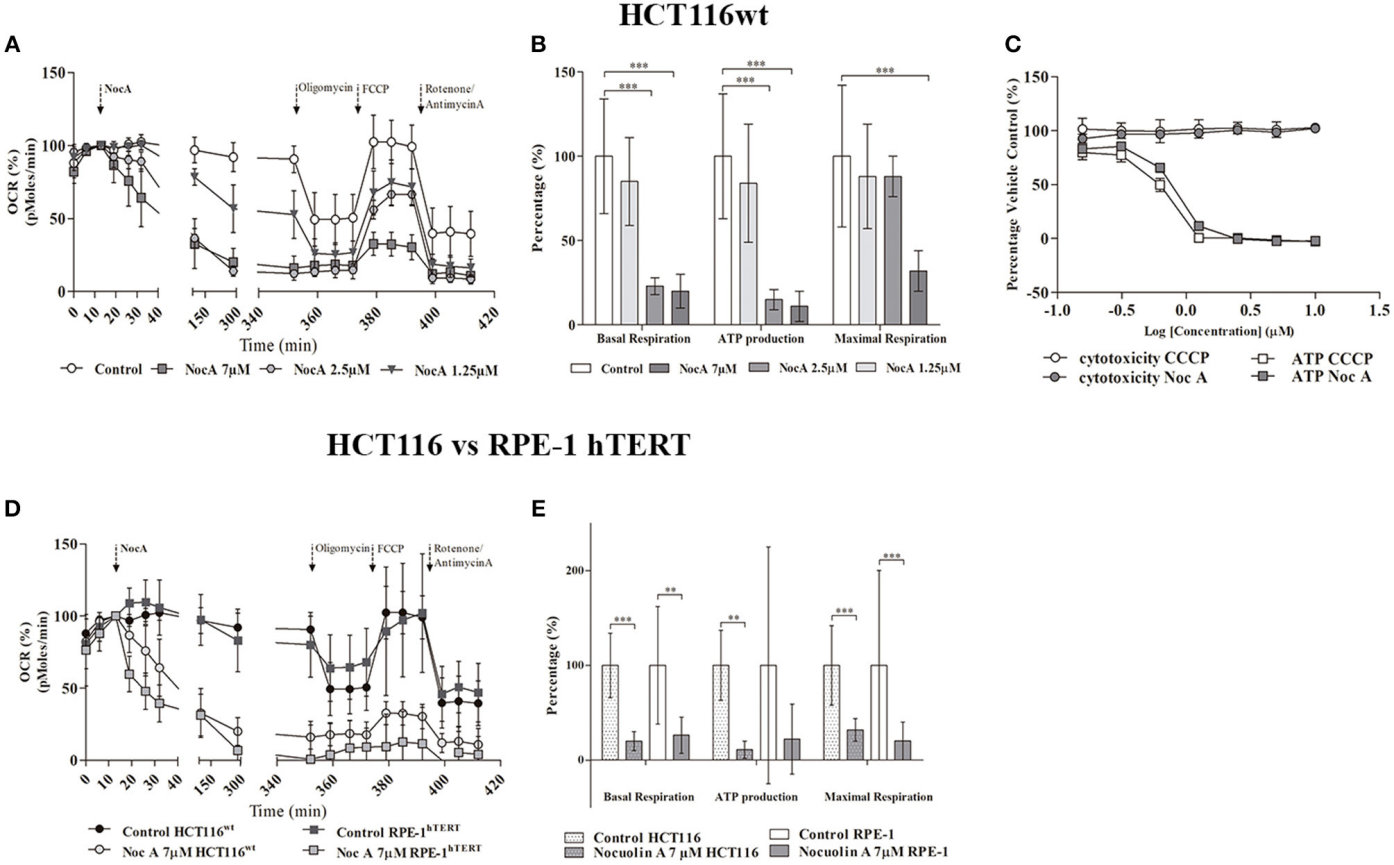
Seahorse analysis of cellular respiration. **(A)** Oxygen consumption rates by HCT116^wt^ cells over 6 h in response to exposure to NocA at 1.25, 2.5, and 7 μM. **(B)** Mitochondrial key parameters—Basal respiration, ATP production, Maximal Respiration—calculated after addition of sequential kit reagents (Oligomycin, FCCP, Antimycin A, and Rotenone) from Seahorse XF Cell Mito Stress Test, Kit ^***^p < 0.001. **(C)** Dose-response for glucose-free (galactose supplemented) HCT116^wt^ cells treated with NocA and CCCP and respective cell viability after 2 h of exposure (according to Mitochondrial ToxGlo™ Assay kit). 1 = Log (10 μM) to −0.81 = Log (0.16 μM). **(D)** Comparison of Oxygen consumption rates between HCT116^wt^ and RPE-1 hTERT cells in response to 6 h exposure to NocA at 7 μM. **(E)** Mitochondrial key parameters—Basal respiration, ATP production, Maximal Respiration- calculated after using Seahorse XF Cell Mito Stress Test Kit on RPE-1 hTERT and HCT116^wt^ exposed to NocA 7 μM for 6 h. ^***^p < 0.001; ^**^p < 0.01, *n* > 5, 3 independent assays.

In order to directly examine whether NocA result in a metabolic insufficiency, intracellular ATP concentrations were determined. Decreases of ATP were indeed detected with increasing concentrations of NocA, after 2 h of exposure to concentrations as low as 0.16 μM NocA. The decreases in ATP occurred prior to induction of cytotoxicity and were similar in amplitude to those induced by the mitochondrial uncoupler CCCP (Figure 5C).

The pronounced metabolic effect by NocA raised the possibility that the compound acts as a mitochondrial toxin. Cells cultured in glucose free medium (supplemented with galactose) showed an increase of sensitivity to NocA, supporting the idea of mitochondrial toxicity (Table 4). Mitochondrial respiration rates were measured using a Seahorse Extracellular Flux Analyzer. Cells were exposed to different concentrations of NocA and oxygen consumption rates were recorded over 6 h (Figure 5A). The basal respiration, ATP production, and non-mitochondrial respiration were decreased by 7 and 2.5 μM NocA, while maximal respiration was only reduced by 7 μM NocA (Figure 5B). The same analysis was performed with confluent RPE-1 hTERT cells exposed to 7 μM NocA, and similar effects were observed (Figure 5D) on basal respiration, maximal respiration but ATP production was not significantly reduced (Figure 5E).

**Table 4:** IC_50_ comparison between HCT116^wt^ cells grown in DMEM and glucose-free DMEM, supplemented with galactose.

**Cell line**	**Medium**	**IC50 (μM)**
HCT116	Glucose	1.3 ± 0.9
	Galactose	0.4 ± 0.07

The cationic lipophilic fluorochrome dye TMRE (tetramethylrhodamine, ethyl ester) staining of HCT116 cells indicative of depolarization of the mitochondrial membrane, did not show any differences between solvent control and cells exposed to 7 μM NocA (Supplementary Figure 7).

## Discussion

Oxadiazoles and oxadiazines have recently attracted attention due to the exhibition of potential anticancer properties (46–49). We here report that the oxadiazine NocA shows antiproliferative activity on colon cancer cells grown as monolayer cultures and also on MTS. Conventional cancer therapeutic drugs show overall poor efficacy on MTS, a phenomenon which is believed to be reflected in limited efficacy of drugs to act on tumor masses *in vivo*. NocA induce significantly apoptosis on MTS at a concentration (2.5 μM) which is only 2-fold higher than the concentration of IC_50_ *in vitro*. Cisplatin has been reported to induce apoptosis at concentrations >10-fold that of its IC_50_ on monolayer cells (21). The use of cell lines overexpressing BCL2 family proteins revealed that the mechanism of cell death induction is independent of these proteins. Whereas, proliferating HCT116 cancer cells and proliferating RPE-1-hTERT immortalized cells showed similar degrees of sensitivity to NocA, non-proliferating RPE-1-hTERT were less sensitive. These findings are likely to be explained by higher metabolic demands by proliferating cells.

Transcriptome profiling can be used to generate hypotheses with regard to the mechanism of action of compounds (35, 36, 44). The molecular signature of NocA was complex, showing the alteration of a number of cellular responses such as vesicular transport, cellular response to inflammation, protein synthesis inhibitors and ATPase inhibition. Although the strong connectivity of the hits obtained for the described C-Map classes, these pharmacological classes are very distinct, and rather unspecific mechanisms, and did not point to a clear mode of action. The Gene Enrichment Analysis and Gene Ontology (GSEA) analysis indicated stress responses, including endoplasmic reticulum (ER) stress, and also autophagy, which we selected for the following analyses. ER stress was analyzed by p-EIF2α protein expression (50), but no upregulation was observed, which would indicate ER stress (data not shown). The increased expression of the autophagy marker LC3B-II as well as increased MDC staining of monolayer cells and MTS suggested evidence for the induction of autophagy. Future experiments should focus on the extent of autophagy and the analysis of the autophagic flux (51). Here, the indication of autophagy led us to speculate that treatment with NocA may interfere with cellular energy production. Our data confirmed this hypothesis, showing decreases in basal mitochondrial oxygen consumption rates and a rapid decrease in cellular ATP levels. These findings suggest that NocA is a mitochondrial toxin targeting the mitochondrial function of colon cancer cells without alteration of the mitochondrial membrane potential. These results obtained for both HCT116 and RPE-1 reinforce the previous idea that cancer cells are more susceptible to NocA than normal non-proliferating cells due to the higher energy requirements and proliferating characteristics. In contrast, the cyanobacterial compounds hierridin B and portoamides AB isolated by our lab targeted mitochondrial function by manipulation of mitochondrial membrane potential (18, 19).

Targeting bioenergetic balance of cancer cells is attracting significant attention as a strategy to treat cancer (52, 53). The concept of targeting mitochondria was proposed by Lan Bo Chen in the 1980s (54, 55) and was later described also by Fantin et al. (56). More recently, the interest in mitochondria as therapeutic targets have increased due to the demonstration of increased mitochondrial function in cancer stem cells (57) and the finding that quiescent tumor cells are dependent on oxidative phosphorylation (32, 52, 58). The finding that NocA is effective on MTS is consistent with the effects on mitochondrial OXPHOS. Quiescent cells in hypoxic and nutritionally compromised microenvironments in MTS are expected to show a limited metabolic plasticity and to be particularly sensitive to inhibition of the major source of ATP production, i.e., OXPHOS (52). The increased sensitivity to glucose starvation observed during NocA treatment is consistent with a state of decreased metabolic plasticity and vulnerability of energy production.

We conclude that the antiproliferative mechanism of NocA is likely to be due to mitochondrial inhibition. Whether NocA can be developed as anticancer compound is unclear. Future studies should focus on systemic toxicity of NocA, as well as on evaluation of cancer selectivity and pharmacological factors such as metabolism and distribution.

## Data Availability

This manuscript contains previously unpublished data. The name of the repository and accession number are not available.

## Author Contributions

MS, VV, SL, and RU contributed to the conceptualization of the research. MS, MP, SL, and RU contributed to the formal analysis. RU and VV contributed to the funding acquisition. MS, MP, VV, SL, and RU contributed to the investigation and the development of methodology and validated the work. SL and RU provided supervision of the work. MS, SL, and RU wrote the original paper.

### Conflict of Interest Statement

The authors declare that the research was conducted in the absence of any commercial or financial relationships that could be construed as a potential conflict of interest.
