# Corrigendum: Antiproliferative effects of the natural oxadiazine Nocuolin A are associated with impairment of mitochondrial oxidative phosphorylation

**DOI:** 10.3389/fonc.2023.1228436

**Published:** 2023-06-02

**Authors:** Maria Lígia Sousa, Marco Preto, Vítor Vasconcelos, Stig Linder, Ralph Urbatzka

**Affiliations:** ^1^Faculty of Sciences of University of Porto, Porto, Portugal; ^2^Interdisciplinary Centre of Marine and Environmental Research, Porto, Portugal; ^3^Department of Oncology and Pathology, Cancer Centre Karolinska, Karolinska Institute, Stockholm, Sweden; ^4^Department of Medical and Health Sciences, Linköping University, Linköping, Sweden

**Keywords:** natural products, cyanobacteria, spheroids, colon cancer, anti-cancer drugs, mitochondria, autophagy

In the published article, a reference in the funding statement was erroneously omitted as published. Instead of:

This research was supported by the Structured Program of R&D&I INNOVMAR—Innovation and Sustainability in the Management and Exploitation of Marine Resources (reference NORTE-01-0145-FEDER-000035, Research Line NOVELMAR), funded by the Northern Regional Operational Program (NORTE2020) through the European Regional Development Fund (ERDF). The project was additionally supported the project CYANCAN—Uncovering the cyanobacterial chemical diversity: the search for novel anticancer compounds **(reference PTDC/MEDQUI/30944/2017)** co-financed by NORTE 2020, Portugal 2020, and the European Union through the ERDF, and by Foundation for Science and Technology through national funds. RU was supported by the FCT postdoc grant SFRH/BPD/112287/2015 and MS by the FCT PhD grant SFRH/BD/108314/2015.

**It should be:**

This research was supported by the Structured Program of R&D&I INNOVMAR—Innovation and Sustainability in the Management and Exploitation of Marine Resources (reference NORTE-01-0145-FEDER-000035, Research Line NOVELMAR), funded by the Northern Regional Operational Program (NORTE2020) through the European Regional Development Fund (ERDF). The project was additionally supported the project CYANCAN—Uncovering the cyanobacterial chemical diversity: the search for novel anticancer compounds **(reference PTDC/MED-QUI/30944/2017 - NORTE-01-0145-FEDER-030944)** co-financed by NORTE 2020, Portugal 2020, and the European Union through the ERDF, and by Foundation for Science and Technology through national funds.

The authors apologize for this error and state that this does not change the scientific conclusions of the article in any way. The original article has been updated.

