# Supplementary material for: Antiproliferative Effects of the Natural Oxadiazine Nocuolin A Are Associated With Impairment of Mitochondrial Oxidative Phosphorylation
Source: Front Oncol. 2019 Apr 3;9:224. doi: 10.3389/fonc.2019.00224 (PMC6456697; doi:10.3389/fonc.2019.00224)
Supplement: Supplementary file 3 [file Data_Sheet_1.docx]

**Supplementary material**

**The natural oxadiazine Nocuolin A kills cancer cells by impairing OXPHOS without disruption of mitochondrial membrane potential**


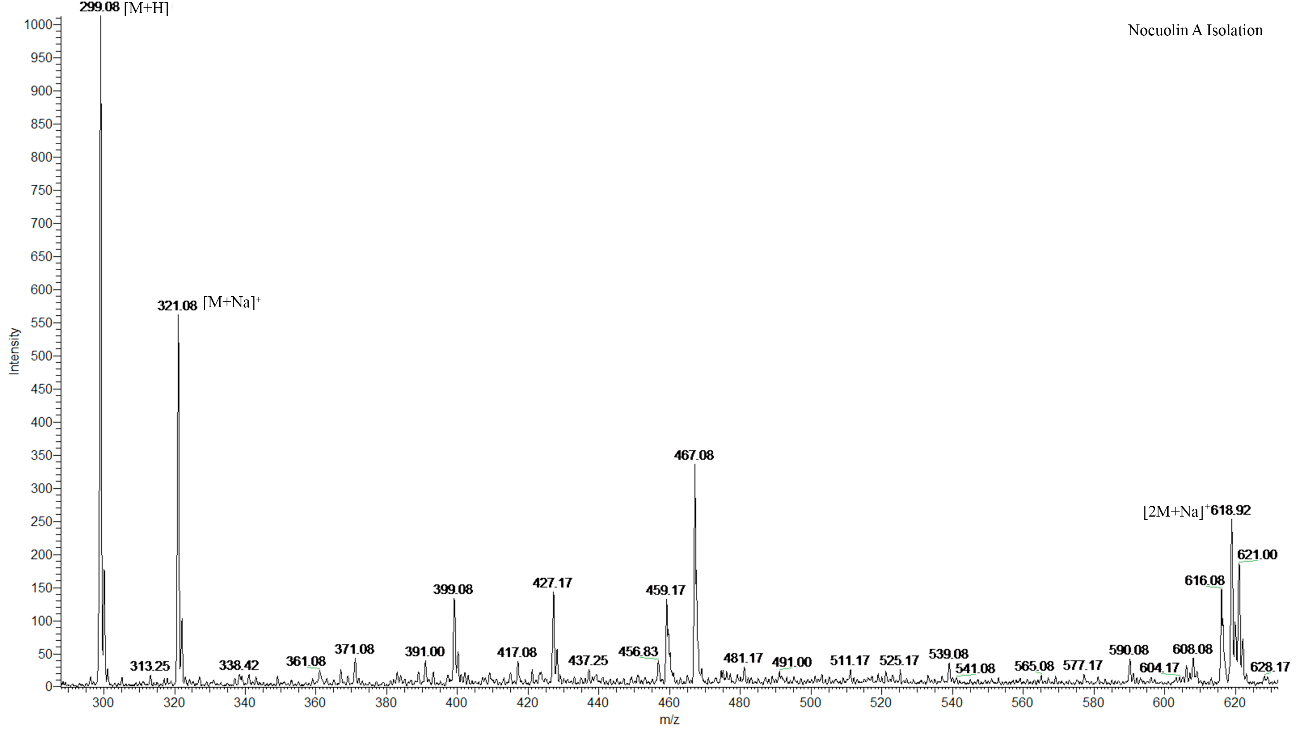


Supplementary figure 1 – HRMS spectra of Nocuolin A isolation.


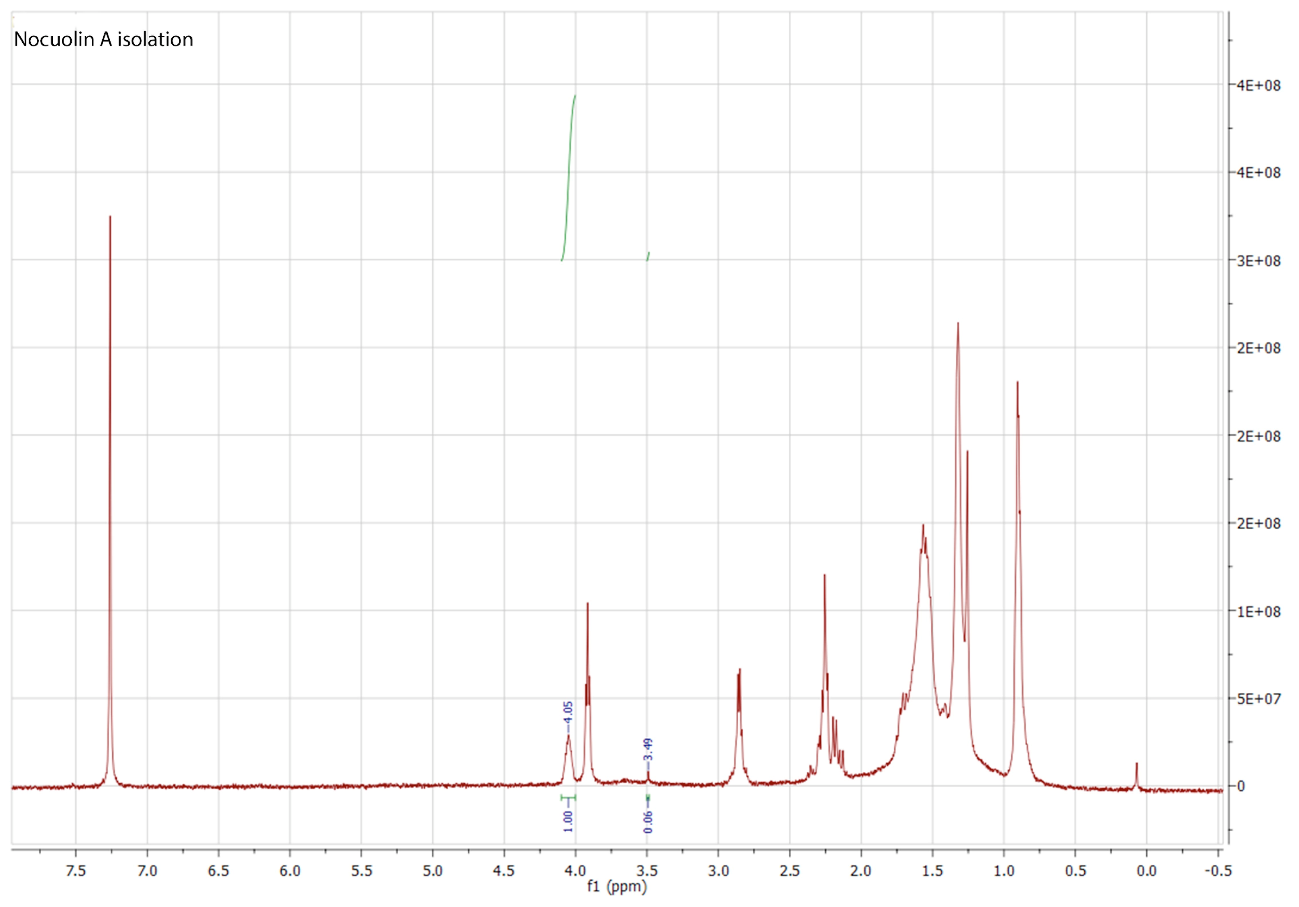
Supplementary figure 2 - 1H NMR (400 MHz) spectra of Nocuolin A isolation in chloroform-d.


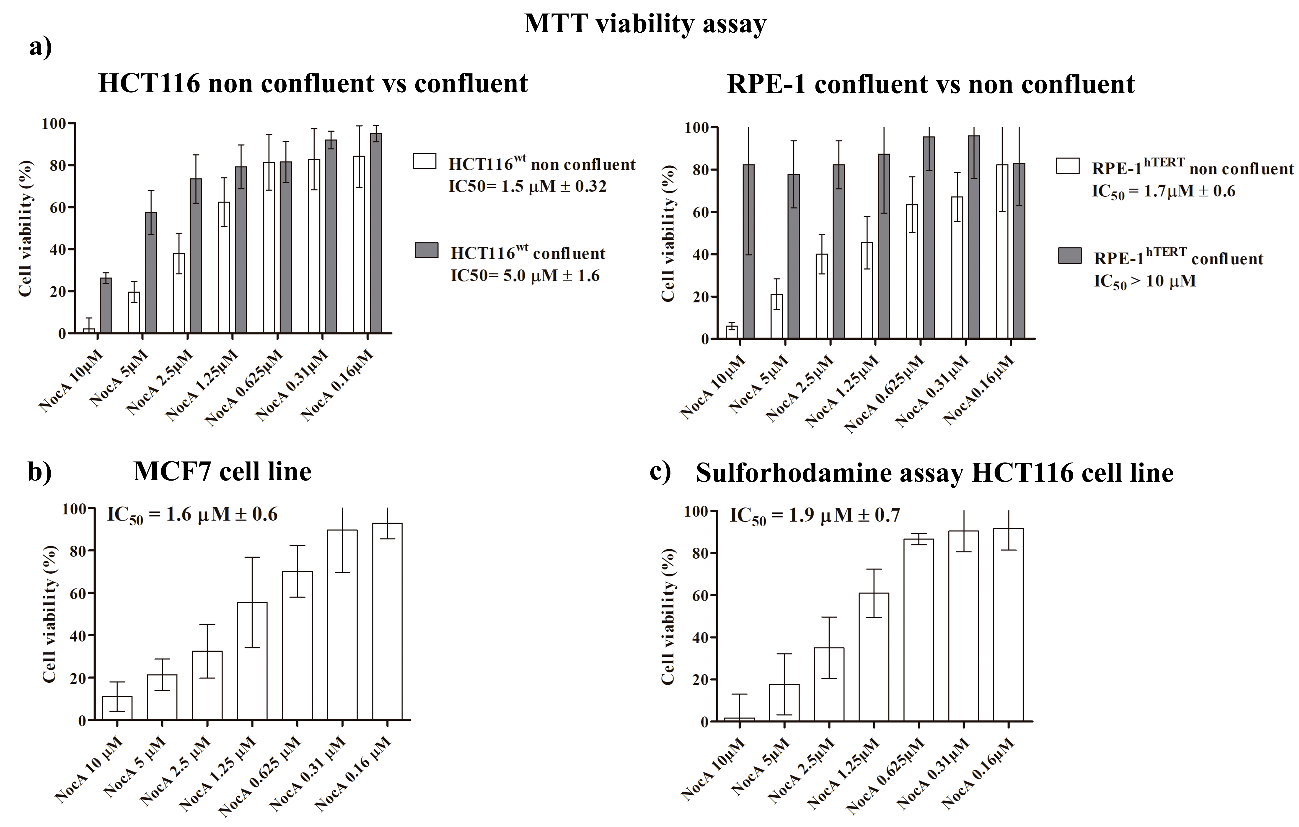


Supplementary figure 3 – Viability assays performed on the cell lines studied. Panel a) MTT viability assay performed on Monolayer HCT116 cell line and RPE-1 hTERT cell line. Left graph shows HCT116 cell cultured exhibited 80% confluence at the end of the experiment on control wells (non-confluent culture) in comparison with HCT116 cell culture at 100% confluence at the end of the experiment (confluent culture). n>3, two independent assays where the confluent and non-confluent assay were performed simultaneously. Right graph with the same assay performed on monolayer hTERT-RPE-1 cell line. n>4, three independent assays. Panel b) IC50 on MCF7 cell line based on MTT assay. n= 8, 3 independent experiments. c) Sulforhodamine viability assay confirming the MTT assay results. n>3, two independent assays.


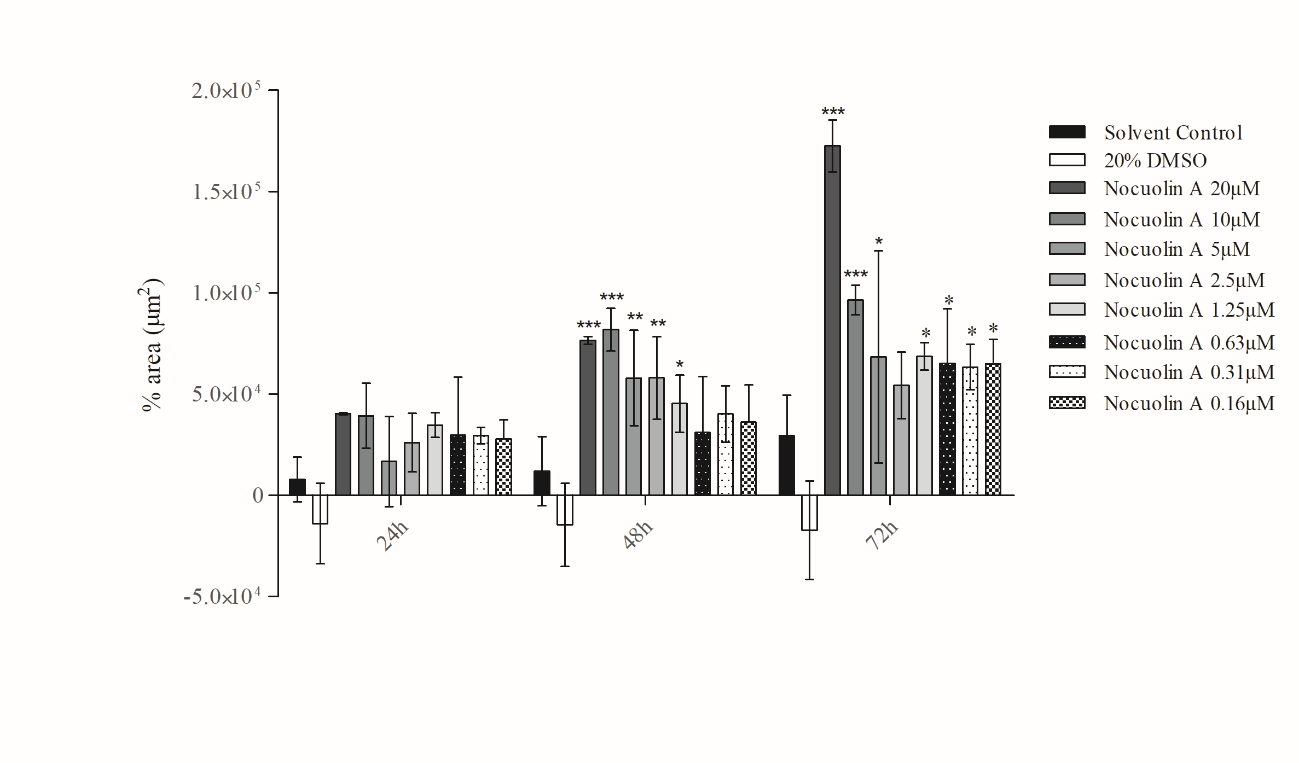
Supplementary Figure 4 - Alteration of MTS size during exposure to NocA over 72h. The measurement was performed based on the images retrieved from the IncuCyte™ ZOOM Analysis System (Essen BioScience, Inc.). The increasing size of the spheroid reveals a disaggregation of the structure due to cell death. n>3. This assay was repeated using HCT116 with red nucleus with n=6. Error bars stand for standard deviation. * p<0.05; ** p<0.01; *** p<0.001.

*
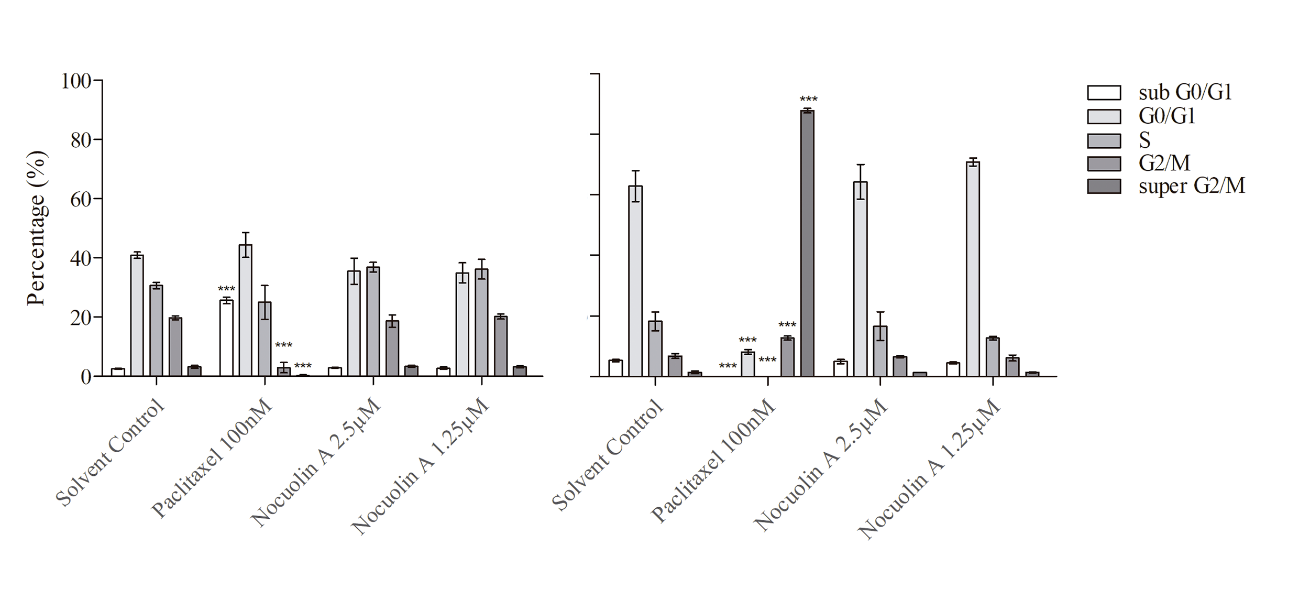
*Supplementary figure *5* - Cell Cycle statistics. No significant alterations were observed on cells exposed to NocA. Error bars represent standard deviation. (*** p<0.01), at 24h or 48h. 10 000 events counted per gated replicate, n=3.


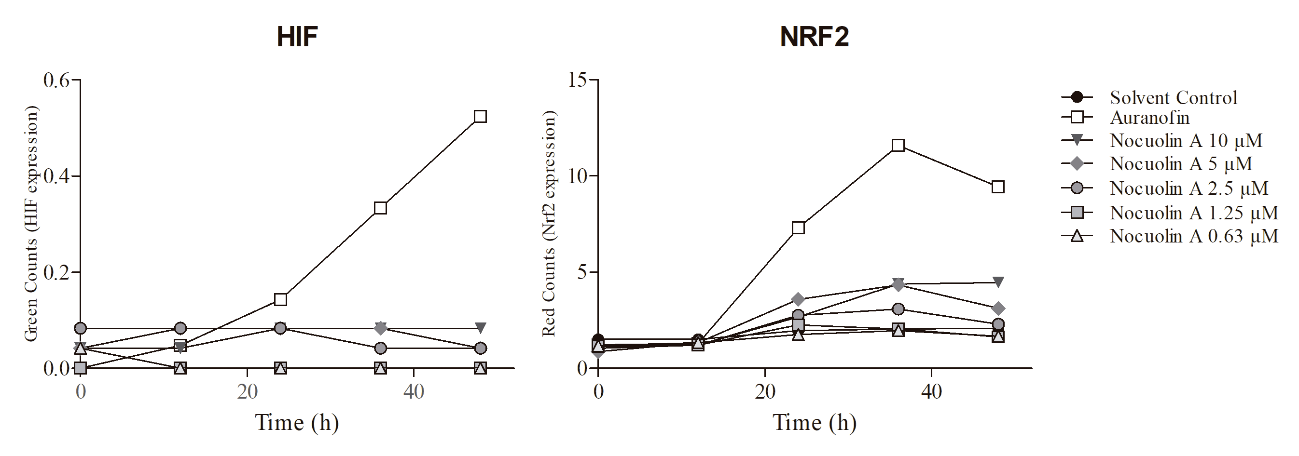
Supplementary figure 6 - NocA does not induce Nrf2 or HIF expression on HCT116 over 48h of exposure. a) Auranofin was used as positive control. b) VLX600 was used as positive control. Graphs generated by IncuCyte™ ZOOM Analysis System (Essen BioScience, Inc.). n=8.


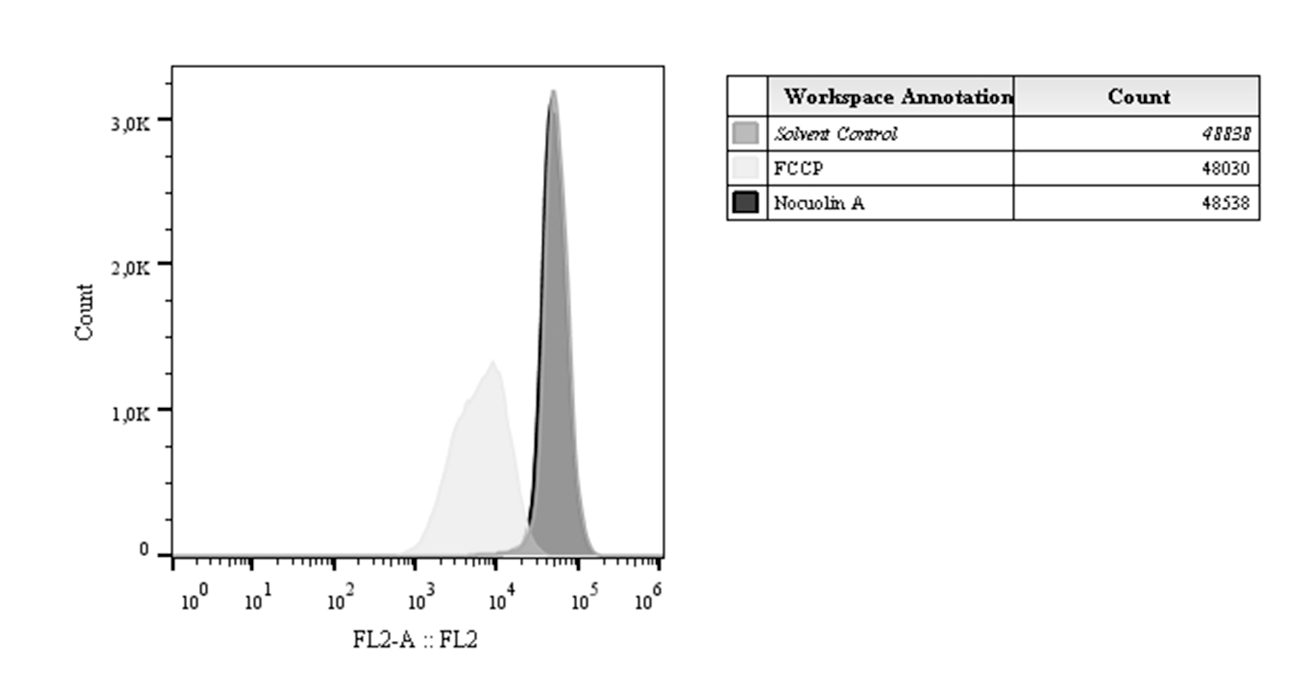
Supplementary figure 7 – TMRE flow cytometry fluorescence on polarized mitochondria. FCCP as positive control. No significant alterations on fluorescence were observed on cells exposed to NocA 7µM for 6h. 60 000 events per sample, n=2.

Supplementary table 1 - Go Terms and respective Genes ID responsible to its enrichment.

| **Cluster** | **Go Term** | **N.º of genes** | **Genes ID** |
| --- | --- | --- | --- |
| Immune Response | GO_RESPONSE_TO_BACTERIUM | 243 | PPM1D\|HLA-E\|RPL39\|IGHA1\|HIST1H2BG\|HIST1H2BJ\|HIST1H2BK\|HIST1H2BI\|HLA-A\|HIST1H2BF\|HIST2H2BE\|HIST1H2BC\|CAV1\|SEH1L\|PRKCD\|IL8\|FCER1G\|PDE4B\|SYK\|S100A9\|XBP1\|STAT5B\|DAB2IP\|FOS\|TH\|EIF2AK2\|OPTN\|ERAP1\|RNF5\|CCL2\|LITAF\|TLR2\|NFKBIA\|WASL\|NFKBIB\|ZC3H12A\|IL27RA\|VLDLR\|SCARB1\|ABCA1\|IGHE\|CCL5\|CCL20\|BYSL\|ICAM1\|IRAK1\|RELA\|IRAK2\|PPARD\|CCL3\|APP\|IKBKB\|PRB3\|PTGER3\|HLA-B\|P2RY2\|LEAP2\|LYN\|PAWR\|CD47\|LYST\|NFKB1\|CD160\|PLA2G2A\|ANKRD1\|EDN1\|ANXA3\|PTGER4\|RAB14\|HDAC2\|VGF\|TNIP2\|JAK2\|MUC5B\|ANKRD17\|MAPK9\|BAIAP2L1\|SOCS3\|COCH\|FN1\|MR1\|CEBPB\|SNX3\|TRAF6\|ROMO1\|DEFB119\|VIL1\|PLAC8\|DROSHA\|PYCARD\|SERPINB9\|FUCA2\|TMF1\|UPF1\|BAIAP2\|IRF3\|FAS\|ISG15\|IL6\|MAVS\|TBK1\|NOD1\|TICAM1\|CASP8\|NOD2\|PRKCE\|TNFAIP3\|MAPKAPK2\|BCL10\|RPS6KA3\|TLR5\|TNFRSF10B\|MAPKAPK3\|SLC11A1\|JUN\|PLA2G6\|NFKB2\|TNFRSF1A\|NFKBIL1\|TNFRSF18\|HNRNPA0\|TNFRSF21\|IL1B\|HLA-DRB5\|TNFRSF10C\|CXCL5\|S100A14\|CXCL1\|NOTCH1\|C5AR1\|TNF\|F3\|IL23A\|IL6R\|ADAM17\|CXCL2\|RELT\|LTBR\|AKT1\|CXCL3\|TNFRSF10A\|PTAFR\|HMGB2\|SERPINE1\|HCK\|CCR7\|F2R\|HLA-DRB1\|EDNRA\|TNFRSF8\|PTGES\|ASS1\|HDAC5\|CITED1\|MYD88\|TNFRSF11A\|RIPK2\|RBPJ\|SRC\|LIAS\|CPEB1\|ABL1\|ALAD\|CMPK2\|IL18\|CTR9\|TGFB1\|MAPK8\|SRR\|MAPK14\|CXCL16\|BCR\|ABR\|SPON2\|AKIRIN2\|MGST2\|ENTPD2\|NCL\|OTUD5\|RARA\|CDC73\|NRF1\|PALM3\|PTGER2\|MALT1\|ADAM9\|FZD5\|PRPF8\|CASP3\|GFI1\|GCH1\|SOCS1\|TRIB1\|CCRN4L\|PTPN22\|CD80\|SHPK\|DUSP10\|ERBB2IP\|PLSCR4\|PELI1\|NR1D1\|FMO1\|MTDH\|SBNO2\|MGST1\|LOXL1\|PAF1\|TNFRSF11B\|B2M\|TSPO\|THBD\|NR1H3\|FER\|JUND\|PTGFR\|IRF5\|CASP9\|PROS1\|GNG12\|TFAP2A\|IFNAR1\|JUNB\|HNRNPM\|CNP\|MAOB\|KCNJ8\|PLCG2\|MAPK1\|HSF1\|PRDX3\|CDK19\|CYP1A1\|TIMP4\|ADH5\|OGT\|CYP1A2\|BCL3\|FOXP1\|COMT\|RAB1A\|SIRT2\|ADM |
|  | GO_INFLAMMATORY_RESPONSE | 205 | HFE\|SEH1L\|IL8\|FCER1G\|PIK3CD\|SYK\|S100A9\|EMR2\|HRH1\|STAT5B\|GAL\|DAB2IP\|FOS\|NFE2L2\|CCL2\|TLR2\|ZC3H12A\|CCL5\|CCL20\|RXRA\|LIPA\|ICAM1\|APOL2\|RELA\|IRAK2\|CCL26\|RPS6KA5\|CCL3\|IKBKB\|LYN\|NFKB1\|STAT3\|CSF1R\|TNIP2\|CCL4\|CCL22\|FN1\|CEBPB\|RPS6KA4\|ITCH\|PYCARD\|FFAR2\|HYAL1\|FAS\|IL6\|IKBKG\|TBK1\|NOD1\|TICAM1\|CLEC7A\|CASP4\|TNFAIP3\|MAPKAPK2\|TLR5\|TNFRSF10B\|TNIP1\|SLC11A1\|NFKB2\|TNFRSF1A\|REL\|ALOX15\|THBS1\|TNFRSF18\|HNRNPA0\|ANO6\|TNFRSF21\|HMGB1\|IL17RE\|NFRKB\|IL1B\|PRKCZ\|HLA-DRB5\|ACVR1\|RARRES2\|TNFRSF10C\|CYP4F11\|SLC7A2\|CXCL5\|PLA2G4C\|NOTCH1\|CXCL1\|AGER\|C5AR1\|LTA4H\|TNF\|AOX1\|C3AR1\|F3\|IL23A\|NMI\|SMAD1\|ITGB6\|BLNK\|IL6R\|NFKBID\|RASGRP1\|SIGIRR\|CSF1\|EPHX2\|CXCL2\|RELT\|LTBR\|CXCL3\|AKT1\|TNFRSF10A\|PTAFR\|HMGB2\|ABCF1\|ADAM8\|LXN\|CCR7\|HCK\|PARP4\|CCR1\|CAMK1D\|HMOX1\|MGLL\|HNRNPK\|SERPINF2\|UCN\|F2R\|HLA-DRB1\|F2RL1\|SNAP23\|CTNNBIP1\|EIF2AK1\|RAC1\|SERPINA1\|PNMA1\|AFAP1L2\|PRKD1\|TNFRSF8\|NFX1\|OGG1\|NFE2L1\|TOLLIP\|PLAA\|PRDX5\|PTGES\|IL4R\|ADORA1\|TFRC\|ASS1\|PXK\|ECM1\|ATRN\|F12\|MAP2K3\|CYP26B1\|GPR68\|NAIP\|HDAC5\|CD97\|IL1RAP\|NUPR1\|MYD88\|F8\|S1PR3\|IGFBP4\|B4GALT1\|GSDMD\|SDC1\|SPHK1\|NFATC4\|MMP25\|TNFRSF11A\|SEMA7A\|ANXA1\|NDST1\|RIPK2\|RBPJ\|PLSCR1\|UNC13D\|HDAC4\|KRT16\|CCR3\|BCL6\|XCR1\|TPST1\|CXCR4\|BMPR1B\|CCRL2\|F11R\|CHST1\|POLB\|LIAS\|NOX1\|ELF3\|IL18\|IL17C\|TGFB1\|BMP6\|IL1RN\|IL17D\|SP100\|AIMP1\|SCG2\|SPP1\|C5\|BMP2\|TNFRSF11B\|PARK7\|KDM6B\|GATA3\|RELB |
|  | GO_RESPONSE_TO_MOLECULE_OF_BACTERIAL_ORIGIN | 180 | TNFRSF18\|HNRNPA0\|TNFRSF21\|IL1B\|TNFRSF10C\|CXCL5\|S100A14\|CXCL1\|NOTCH1\|C5AR1\|TNF\|F3\|IL8\|PDE4B\|ADAM17\|CXCL2\|RELT\|LTBR\|XBP1\|AKT1\|CXCL3\|PTAFR\|TNFRSF10A\|STAT5B\|HMGB2\|SERPINE1\|HCK\|CCR7\|DAB2IP\|FOS\|TH\|EIF2AK2\|F2R\|EDNRA\|TNFRSF8\|PTGES\|ASS1\|HDAC5\|CCL2\|CITED1\|LITAF\|TNFRSF11A\|TLR2\|RIPK2\|SRC\|NFKBIA\|LIAS\|NFKBIB\|CPEB1\|ABL1\|ALAD\|CMPK2\|IL18\|CTR9\|MAPK8\|TGFB1\|SRR\|ZC3H12A\|MAPK14\|BCR\|CXCL16\|ABR\|SPON2\|AKIRIN2\|VLDLR\|MGST2\|ENTPD2\|SCARB1\|NCL\|ABCA1\|OTUD5\|RARA\|CDC73\|NRF1\|PALM3\|PTGER2\|CCL5\|MALT1\|ADAM9\|CCL20\|BYSL\|FZD5\|ICAM1\|PRPF8\|IRAK1\|CASP3\|GFI1\|RELA\|GCH1\|IRAK2\|SOCS1\|PPARD\|TRIB1\|CCRN4L\|CCL3\|PTPN22\|CD80\|IKBKB\|SHPK\|DUSP10\|ERBB2IP\|PTGER3\|P2RY2\|PLSCR4\|PELI1\|LYN\|PAWR\|NR1D1\|FMO1\|NFKB1\|MTDH\|SBNO2\|ANKRD1\|MGST1\|EDN1\|PTGER4\|LOXL1\|HDAC2\|TNIP2\|JAK2\|PAF1\|TNFRSF11B\|B2M\|MAPK9\|TSPO\|THBD\|SOCS3\|NR1H3\|FER\|FN1\|CEBPB\|JUND\|TRAF6\|PTGFR\|IRF5\|CASP9\|PROS1\|GNG12\|TFAP2A\|PYCARD\|IFNAR1\|JUNB\|UPF1\|HNRNPM\|FAS\|IRF3\|CNP\|IL6\|MAOB\|KCNJ8\|PLCG2\|MAPK1\|HSF1\|PRDX3\|CYP1A1\|CDK19\|TIMP4\|ADH5\|OGT\|TICAM1\|CASP8\|CYP1A2\|NOD2\|PRKCE\|TNFAIP3\|MAPKAPK2\|FOXP1\|BCL10\|COMT\|RPS6KA3\|TLR5\|SIRT2\|TNFRSF10B\|MAPKAPK3\|SLC11A1\|JUN\|NFKB2\|TNFRSF1A\|NFKBIL1\|ADM |
|  | GO_POSITIVE_REGULATION_OF_RESPONSE_TO_EXTERNAL_STIMULUS | 157 | BMPR2\|THBS1\|MAPK13\|SUPT5H\|SQSTM1\|CALCOCO2\|EPM2A\|SCOC\|LARP1\|RALB\|PIP4K2C\|OSMR\|SNX4\|PUM1\|GPRC5B\|VAMP8\|RAB12\|BNIP3\|LRSAM1\|CLOCK\|IL17RB\|ZP3\|PIP4K2B\|ZC3HAV1\|ATG7\|FKBP1B\|NPY5R\|USF1\|ANO6\|WDFY3\|HMGB1\|DAPK2\|AMBRA1\|IL1B\|KIAA1324\|RAB3GAP1\|WAC\|VEGFB\|PUM2\|RARRES2\|ADORA2B\|BNIP3L\|CXCL12\|NDEL1\|CXCL5\|DDX60\|S100A14\|SNW1\|CXCL1\|CALR\|RAB3GAP2\|AGER\|C5AR1\|PAFAH1B2\|TNF\|PIP4K2A\|C3AR1\|PDGFB\|F3\|IL8\|IL23A\|FCER1G\|SWAP70\|F7\|CD74\|IL6R\|ADAM17\|CSF1\|S100A9\|CXCL2\|VEGFC\|EDN2\|CXCL3\|FIGF\|STAT5B\|IL17RA\|ADAM8\|RPS19\|SERPINE1\|CCR7\|CCR1\|CAMK1D\|GAS6\|CXCL14\|SERPINF2\|TRPV4\|ITGA2\|PTK2B\|F2RL1\|EDNRA\|RAC1\|VEGFA\|ADAM10\|PRKD1\|HSPB1\|F12\|CCL2\|PRKCA\|TNFRSF11A\|TLR2\|RIPK2\|CREB3\|IL18\|TGFB1\|BMP6\|TIAM1\|FGF18\|BECN1\|SH3GLB1\|AKT2\|SMAD3\|STX3\|ARTN\|PRKD2\|CCL5\|PPM1F\|STX4\|MET\|FGFR1\|MEGF8\|DSCAM\|SCG2\|CCL26\|CCL3\|PTGER3\|SASH1\|CD47\|SLIT2\|PLA2G2A\|PTGER4\|JAK2\|IL6ST\|ANKRD17\|CCL4\|THBD\|SIRT1\|PARK7\|FN1\|TRAF6\|FFAR2\|IL6\|TBK1\|CDK19\|NOD1\|SPTLC2\|NOD2\|TRIM13\|BRAF\|SPTLC1\|NRP1\|ATG5\|TNIP1\|SEMA5A\|LPAR1\|ULK1\|GBA\|TNFRSF1A |
|  | GO_CELLULAR_RESPONSE_TO_BIOTIC_STIMULUS | 101 | IL1B\|TXNIP\|HSPA5\|TNF\|IL8\|PDE4B\|SYK\|WFS1\|MUS81\|FBXO18\|SLX4\|XBP1\|AKT1\|PTAFR\|HMGB2\|SERPINE1\|HCK\|DAB2IP\|EIF2AK3\|ASS1\|CCL2\|HDAC5\|LITAF\|TLR2\|RIPK2\|SRC\|DDIT3\|NFKBIA\|NFKBIB\|CPEB1\|ABL1\|IL18\|CMPK2\|CTR9\|TGFB1\|MAPK8\|ZC3H12A\|MAPK14\|CXCL16\|SPON2\|VLDLR\|ENTPD2\|SCARB1\|NCL\|ABCA1\|RARA\|CDC73\|TP53\|CCL5\|ADAM9\|CCL20\|FZD5\|BYSL\|PRPF8\|ICAM1\|IRAK1\|GFI1\|RELA\|IRAK2\|PPARD\|PTPN22\|CCL3\|CD80\|SHPK\|PTGER3\|P2RY2\|PLSCR4\|LYN\|NR1D1\|NFKB1\|MTDH\|ANKRD1\|SBNO2\|TNIP2\|PAF1\|B2M\|TSPO\|MAPK9\|NR1H3\|FN1\|CEBPB\|TRAF6\|PYCARD\|HNRNPM\|UPF1\|IRF3\|IL6\|PPP1R15B\|MAPK1\|GSK3B\|ATG10\|CDK19\|OGT\|TICAM1\|PRKCE\|CLEC7A\|TNFAIP3\|APAF1\|TLR5\|SIRT2\|NFKBIL1 |
|  | GO_REGULATION_OF_LEUKOCYTE_MIGRATION | 84 | THBS1\|BCR\|CYP19A1\|ABR\|CCL28\|CCL5\|CCL20\|ICAM1\|CCL3\|ZP3\|FADD\|TNFRSF18\|PTGER3\|ANO6\|C5\|PADI2\|HMGB1\|PAWR\|LYN\|DAPK2\|MPP1\|IL1B\|SLIT2\|LGALS3\|VEGFB\|RARRES2\|MIA3\|CXCL12\|PTGER4\|CXCL5\|S100A14\|ADA\|CXCL1\|CALR\|CCL4\|C5AR1\|TNF\|C3AR1\|IL23A\|IL8\|SWAP70\|F7\|CD74\|IL6R\|ADAM17\|ITGA2B\|PYCARD\|CSF1\|CXCL2\|VEGFC\|EDN2\|CXCL3\|IL6\|PTAFR\|PLCB1\|FIGF\|ADAM8\|SERPINE1\|CCR7\|CCR1\|CAMK1D\|HMOX1\|GAS6\|CXCL14\|MADCAM1\|TRPV4\|ITGA2\|PTK2B\|F2RL1\|EDNRA\|KITLG\|RAC1\|VEGFA\|ADAM10\|ADORA1\|ECM1\|CCL2\|ANXA1\|CREB3\|HOXA7\|APOD\|WASL\|P2RY12\|STK10 |
|  | GO_CELL_CHEMOTAXIS | 79 | CXCL16\|CCL28\|CCL5\|CCL20\|SCG2\|CCL26\|CCL3\|ANO6\|HMGB1\|C5\|IL1B\|TGFB2\|LYST\|LGALS3\|EDN1\|CXCL12\|CXCL5\|CXCL1\|PRKCD\|CCL4\|C5AR1\|CCL22\|PDGFB\|IL8\|GBF1\|FCER1G\|PDE4B\|PIK3CD\|IL6R\|SYK\|PREX1\|S100A9\|FFAR2\|CXCL2\|EMR2\|NUP85\|EDN2\|PIP5K1C\|IL6\|CXCL3\|HRH1\|HMGB2\|ADAM8\|IL17RA\|RPS19\|CCR7\|VAV1\|CCR1\|VAV3\|GAS6\|CXCL14\|RAC1\|VEGFA\|LEF1\|SBDS\|CORO1A\|TRPM4\|PIP5K1A\|ARHGEF16\|BCAR1\|ENG\|CCL2\|EPHA2\|NRP1\|BIN2\|EGR3\|RHOG\|PARVA\|RAB13\|SEMA5A\|NR4A1\|DOCK4\|HBEGF\|TNFRSF11A\|ELMO2\|ANXA1\|LPAR1\|ARRB2\|CXCR4 |
|  | GO_CYTOKINE_ACTIVITY | 73 | IL18\|IL17C\|TGFB1\|TIMP1\|BMP8B\|CXCL16\|CTF1\|BMP6\|CMTM8\|CCL28\|THNSL2\|AREG\|IL1RN\|TNFSF13B\|TNFSF10\|BMP8A\|IL17D\|BMP4\|CCL5\|SP100\|CMTM4\|CCL20\|AIMP1\|TXLNA\|TGFB3\|SCG2\|CMTM3\|GDF15\|CCL26\|CRLF1\|IL7\|CCL3\|LTB\|SPP1\|IL32\|BMP1\|INHBE\|HMGB1\|C5\|LIF\|IL1B\|TGFB2\|IL11\|BMP2\|BMP7\|EDN1\|GRN\|CXCL12\|INHBB\|CXCL5\|TNFRSF11B\|CMTM7\|CXCL1\|CCL4\|TNF\|CCL22\|IL8\|IL23A\|CSF1\|CXCL2\|IL6\|CXCL3\|CXCL14\|KITLG\|VEGFA\|CCL2\|TNFSF9\|CMTM6\|NAMPT\|SECTM1\|FAM3C\|INHA\|GDF11 |
|  | GO_POSITIVE_REGULATION_OF_CHEMOTAXIS | 72 | BMPR2\|TGFB1\|THBS1\|TIAM1\|FGF18\|AKT2\|SMAD3\|STX3\|ARTN\|PRKD2\|CCL5\|PPM1F\|STX4\|MET\|FGFR1\|MEGF8\|DSCAM\|SCG2\|CCL3\|ANO6\|HMGB1\|DAPK2\|IL1B\|SLIT2\|VEGFB\|RARRES2\|CXCL12\|CXCL5\|S100A14\|CALR\|CXCL1\|CCL4\|C5AR1\|PDGFB\|C3AR1\|FN1\|F3\|IL8\|IL23A\|SWAP70\|F7\|CD74\|IL6R\|ADAM17\|CSF1\|CXCL2\|VEGFC\|EDN2\|CXCL3\|IL6\|FIGF\|SERPINE1\|CCR7\|CCR1\|CAMK1D\|GAS6\|CXCL14\|TRPV4\|ITGA2\|PTK2B\|F2RL1\|EDNRA\|RAC1\|VEGFA\|ADAM10\|PRKD1\|HSPB1\|CCL2\|NRP1\|SEMA5A\|LPAR1\|CREB3 |
|  | GO_CELL_ACTIVATION_INVOLVED_IN_IMMUNE_RESPONSE | 71 | STXBP3\|EIF2AK4\|ICAM1\|MLH1\|NR4A3\|NBN\|VAMP8\|CCL3\|ERCC1\|TSC1\|EOMES\|LIG4\|HMGB1\|LYN\|MSH2\|SBNO2\|ANXA3\|PTGER4\|RNF8\|RNF168\|RC3H1\|EXOSC6\|ADA\|EXO1\|GBF1\|FCER1G\|SWAP70\|PIK3CD\|RORA\|SYK\|RASGRP1\|PYCARD\|IL6\|PLCG2\|PTK2B\|F2RL1\|SNAP23\|TICAM1\|SEMA4A\|PRKCE\|ATP7A\|CLEC7A\|GATA3\|BCL3\|HSPD1\|LEF1\|STAT6\|RORC\|BATF\|ZFPM1\|RELB\|FOXP1\|CORO1A\|RC3H2\|PI4K2A\|LAT2\|GATA2\|PLA2G3\|ITFG2\|LFNG\|NOTCH2\|RAB27A\|LGALS1\|SLC11A1\|LCP1\|RPS6\|PSEN1\|DLL1\|UNC13D\|C8ORF4\|ABL1 |
|  | GO_ADAPTIVE_IMMUNE_RESPONSE_BASED_ON_SOMATIC_RECOMBINATION_OF_IMMUNE_RECEPTORS_BUILT_FROM_IMMUNOGLOBULIN_SUPERFAMILY_DOMAINS | 69 | IL18\|TGFB1\|IGHA1\|SLA2\|BTN3A2\|IGHE\|MASP2\|C8G\|CTSH\|MLH1\|ICAM1\|CD46\|HLA-DQB1\|NBN\|JAG1\|HRAS\|DLG1\|EMP2\|SUSD4\|ZP3\|ERCC1\|GNL1\|CTSC\|HMGB1\|LIG4\|C5\|DENND1B\|BTN3A3\|MSH2\|HLA-DRB5\|CD8A\|CADM1\|CD55\|RNF8\|RNF168\|EXOSC6\|MICB\|PRKCD\|EXO1\|C3AR1\|SWAP70\|FCER1G\|TRAF6\|CD74\|RORA\|ADAM17\|FAS\|IL6\|HLA-DRB1\|RFTN1\|SEMA4A\|GATA3\|HSPD1\|BCL3\|LEF1\|CLU\|STAT6\|RORC\|BATF\|IL4R\|FOXP3\|RELB\|BCL10\|IRF7\|RAB27A\|SLC11A1\|UNC13D\|BCL6\|NFKB2 |
|  | GO_RESPONSE_TO_INTERLEUKIN_1 | 66 | ZC3H12A\|VLDLR\|CCL5\|IL1R1\|CCL20\|BYSL\|ICAM1\|IRAK1\|SOX9\|RELA\|IRAK2\|CCL26\|RPS6KA5\|CCL3\|PPP4C\|IKBKB\|RBMX\|PTGER3\|P2RY2\|PAWR\|EGR1\|NFKB1\|PSMB9\|ANKRD1\|EDN1\|PTGER4\|TNIP2\|RC3H1\|MAPK9\|CCL4\|CCL22\|FN1\|CEBPB\|IL8\|TRAF6\|RPS6KA4\|KLF2\|RORA\|HSPA9\|PYCARD\|NKX3-1\|HYAL1\|UPF1\|FAS\|IL6\|PLCB1\|FIGF\|DAB2IP\|CCL2\|TAF9\|CITED1\|IGBP1\|MYD88\|PRKCI\|PRKCA\|YTHDC2\|AES\|CREBBP\|SPHK1\|HNMT\|TNFRSF11A\|ANXA1\|RIPK2\|SRC\|MTHFR\|HDAC4 |
|  | GO_POSITIVE_REGULATION_OF_LEUKOCYTE_MIGRATION | 64 | THBS1\|CCL5\|CCL20\|ICAM1\|CCL3\|ZP3\|FADD\|TNFRSF18\|ANO6\|PTGER3\|HMGB1\|DAPK2\|PAWR\|IL1B\|LGALS3\|VEGFB\|RARRES2\|MIA3\|CXCL12\|PTGER4\|CXCL5\|S100A14\|CXCL1\|CALR\|CCL4\|C5AR1\|TNF\|C3AR1\|IL23A\|IL8\|SWAP70\|F7\|CD74\|IL6R\|ADAM17\|PYCARD\|ITGA2B\|CSF1\|CXCL2\|VEGFC\|EDN2\|IL6\|CXCL3\|PTAFR\|FIGF\|ADAM8\|SERPINE1\|CCR7\|CCR1\|CAMK1D\|GAS6\|CXCL14\|MADCAM1\|TRPV4\|ITGA2\|PTK2B\|F2RL1\|EDNRA\|KITLG\|RAC1\|VEGFA\|ADAM10\|CCL2\|CREB3 |
|  | GO_REGULATION_OF_B_CELL_ACTIVATION | 55 | PPP2R3C\|MMP14\|NDFIP1\|TGFB1\|NFATC2\|CDKN1A\|APLF\|BCL2\|ATM\|IL27RA\|CD81\|SUPT6H\|IGHA1\|PCID2\|SLC39A10\|MZB1\|ATP11C\|TNFSF13B\|TCF3\|UNG\|WHSC1\|SLA2\|IGHE\|PTPN6\|AHR\|PKN1\|ID2\|CASP3\|BANK1\|PAXIP1\|CDKN2A\|THOC1\|IL7\|TNFRSF21\|PAWR\|PELI1\|LYN\|TNIP2\|ADA\|RC3H1\|CD74\|SYK\|XBP1\|FAS\|IL6\|STAT5B\|VAV3\|TICAM1\|NOD2\|STAT6\|TNFAIP3\|FOXP3\|BCL6\|INHA\|IRS2 |
|  | GO_REGULATION_OF_LEUKOCYTE_CHEMOTAXIS | 52 | THBS1\|CYP19A1\|CCL5\|CCL3\|ANO6\|PADI2\|HMGB1\|C5\|LYN\|DAPK2\|IL1B\|MPP1\|SLIT2\|VEGFB\|RARRES2\|CXCL12\|CXCL5\|S100A14\|CXCL1\|CALR\|C5AR1\|CCL4\|C3AR1\|IL23A\|IL8\|SWAP70\|F7\|CD74\|IL6R\|ADAM17\|CSF1\|CXCL2\|VEGFC\|EDN2\|IL6\|CXCL3\|FIGF\|SERPINE1\|CCR7\|CCR1\|CAMK1D\|GAS6\|CXCL14\|TRPV4\|PTK2B\|F2RL1\|EDNRA\|RAC1\|VEGFA\|ADAM10\|CCL2\|CREB3 |
|  | GO_HUMORAL_IMMUNE_RESPONSE | 50 | BCL2\|HLA-E\|RPL39\|SPON2\|IGHA1\|HIST1H2BG\|HIST1H2BJ\|HIST1H2BK\|HIST1H2BI\|HLA-A\|HIST1H2BF\|HIST2H2BE\|HIST1H2BC\|IGHE\|MASP2\|C8G\|CD46\|HLA-DQB1\|IL7\|SUSD4\|ZP3\|APP\|TNFRSF21\|C5\|HLA-DRB5\|CD55\|B2M\|NOTCH1\|EXO1\|TNF\|BLNK\|IL6\|TRAF3IP2\|TFE3\|ST6GAL1\|MNX1\|PAX5\|CFD\|YTHDF2\|TFEB\|CD83\|HLA-DRB1\|BST2\|GATA3\|BCL3\|CLU\|CCL2\|RBPJ\|PLA2G6\|ADM |
|  | GO_MYELOID_LEUKOCYTE_ACTIVATION | 49 | TGFB1\|STXBP3\|CCL5\|ADAM9\|NR4A3\|VAMP8\|CCL3\|HMGB1\|LYN\|SBNO2\|ANXA3\|SLC7A2\|PRKCD\|IL8\|TRAF6\|FCER1G\|JMJD6\|PIK3CD\|SYK\|PYCARD\|PREX1\|RASGRP1\|CSF1\|LTBR\|EDN2\|SNAP23\|F2RL1\|TICAM1\|ADAM10\|PRKCE\|CLU\|BATF\|RELB\|FOXP1\|NDRG1\|LCP2\|CRTC3\|PI4K2A\|LAT2\|GATA2\|TGFBR2\|PLA2G3\|DHRS2\|RHOH\|SLC11A1\|PSEN1\|JUN\|RBPJ\|TNFSF9 |
|  | GO_CELLULAR_RESPONSE_TO_INTERLEUKIN_1 | 48 | ZC3H12A\|DAB2IP\|VLDLR\|CCL5\|IL1R1\|CCL20\|BYSL\|ICAM1\|IRAK1\|SOX9\|RELA\|IRAK2\|CCL26\|RPS6KA5\|CCL3\|PPP4C\|IKBKB\|RBMX\|PTGER3\|P2RY2\|PAWR\|CCL2\|EGR1\|NFKB1\|PSMB9\|ANKRD1\|EDN1\|PTGER4\|TNIP2\|RC3H1\|MAPK9\|CCL4\|CCL22\|FN1\|IL8\|CEBPB\|TRAF6\|RPS6KA4\|KLF2\|RORA\|HSPA9\|PYCARD\|NKX3-1\|HYAL1\|UPF1\|FAS\|IL6\|PLCB1 |
|  | GO_REGULATION_OF_CYTOKINE_BIOSYNTHETIC_PROCESS | 47 | HMOX1\|THBS1\|UBE2J1\|TICAM1\|HSPB1\|TNFRSF8\|GATA3\|BCL3\|CCL20\|IGF2BP1\|FOXP3\|RELA\|MAPKAPK2\|ZNF287\|ZFPM1\|LAG3\|GLMN\|ASB1\|EREG\|MAST2\|IRF1\|LTB\|MAP2K5\|CD276\|BCL10\|CD80\|ERRFI1\|CEBPG\|MAP2K3\|RNF128\|IGF2BP2\|TRIB2\|IL1B\|NFKB1\|KLF4\|INHBB\|TNF\|CEBPB\|NMI\|TRAF6\|SYK\|SIGIRR\|IL6\|PTAFR\|STAT5B\|INHA\|TBK1 |
|  | GO_POSITIVE_REGULATION_OF_LEUKOCYTE_CHEMOTAXIS | 46 | CCR1\|CAMK1D\|GAS6\|CXCL14\|THBS1\|TRPV4\|PTK2B\|F2RL1\|EDNRA\|RAC1\|VEGFA\|ADAM10\|CCL5\|CCL3\|ANO6\|HMGB1\|CCL2\|DAPK2\|IL1B\|VEGFB\|RARRES2\|CXCL12\|CXCL5\|S100A14\|CXCL1\|CALR\|C5AR1\|CCL4\|C3AR1\|IL23A\|IL8\|SWAP70\|F7\|CD74\|IL6R\|ADAM17\|CSF1\|CXCL2\|VEGFC\|CREB3\|EDN2\|IL6\|CXCL3\|FIGF\|SERPINE1\|CCR7 |
|  | GO_MYELOID_LEUKOCYTE_MIGRATION | 44 | VAV3\|RAC1\|VEGFA\|CCL5\|CCL20\|SCG2\|CCL26\|CCL3\|CCL2\|DAPK2\|TGFB2\|IL1B\|LGALS3\|EDN1\|CCL4\|C5AR1\|JAGN1\|CCL22\|TNFRSF11A\|PDGFB\|ANXA1\|GBF1\|IL8\|FCER1G\|PDE4B\|MITF\|PIK3CD\|IL6R\|SYK\|PREX1\|S100A9\|IRAK4\|EMR2\|EDN2\|NUP85\|CXCL3\|PIP5K1C\|IL6\|HRH1\|STAT5B\|IL17RA\|RPS19\|CCR7\|VAV1 |
|  | GO_GRANULOCYTE_MIGRATION | 34 | VAV3\|VEGFA\|CCL5\|CCL20\|SCG2\|CCL26\|CCL3\|CCL2\|DAPK2\|IL1B\|TGFB2\|LGALS3\|EDN1\|C5AR1\|CCL4\|CCL22\|JAGN1\|IL8\|ANXA1\|GBF1\|FCER1G\|PDE4B\|PIK3CD\|SYK\|PREX1\|S100A9\|IRAK4\|EMR2\|EDN2\|PIP5K1C\|CXCL3\|HRH1\|IL17RA\|VAV1 |
|  | GO_POSITIVE_REGULATION_OF_MYELOID_LEUKOCYTE_DIFFERENTIATION | 34 | CCR1\|OGT\|FOS\|CA2\|RIPK1\|RUNX1\|ACIN1\|CD101\|CTNNBIP1\|ATP6AP1\|KITLG\|TMEM64\|CASP8\|HAX1\|GNAS\|TESC\|RB1\|PPARGC1B\|CCL5\|LEF1\|ID2\|TRIB1\|FADD\|GPR68\|CREB1\|KLF10\|LIF\|CSF1R\|PRKCA\|TNF\|IL23A\|TRAF6\|JUN\|CSF1 |
|  | GO_POSITIVE_REGULATION_OF_B_CELL_ACTIVATION | 32 | VAV3\|PPP2R3C\|MMP14\|TGFB1\|CDKN1A\|NFATC2\|BCL2\|CD81\|IGHA1\|PCID2\|SLC39A10\|TICAM1\|ATP11C\|TCF3\|TNFSF13B\|UNG\|NOD2\|WHSC1\|IGHE\|STAT6\|PAXIP1\|IL7\|PELI1\|TNIP2\|ADA\|CD74\|BCL6\|SYK\|XBP1\|IL6\|STAT5B\|IRS2 |
|  | GO_RESPONSE_TO_ANTIBIOTIC | 31 | SKIL\|CYP1A1\|CDKN1B\|UQCRFS1\|PLA2G4F\|PPP2CB\|SLC9A1\|CASP8\|TP53\|CASP3\|HSP90AA1\|EP300\|CCL2\|IL1B\|HSPA5\|PPP1R15A\|JAK2\|SOD1\|CASP9\|MDM2\|JAK1\|HYAL1\|ID1\|UROS\|IL6\|HMBS\|CYB5R4\|RAB10\|RSRC1\|ZC3H8\|ENDOG |
|  | GO_ACUTE_INFLAMMATORY_RESPONSE | 30 | HNRNPK\|SERPINF2\|HFE\|CTNNBIP1\|EIF2AK1\|SERPINA1\|IL1RN\|GATA3\|OGG1\|ICAM1\|PTGES\|APOL2\|TFRC\|ASS1\|F12\|ANO6\|IL1B\|NUPR1\|STAT3\|F8\|ACVR1\|B4GALT1\|FN1\|CEBPB\|F3\|PLSCR1\|IL6R\|SIGIRR\|IL6\|STAT5B |
|  | GO_T_CELL_ACTIVATION_INVOLVED_IN_IMMUNE_RESPONSE | 28 | F2RL1\|SEMA4A\|ATP7A\|EIF2AK4\|GATA3\|BCL3\|LEF1\|STAT6\|RORC\|ICAM1\|BATF\|ZFPM1\|RELB\|FOXP1\|RC3H2\|TSC1\|EOMES\|HMGB1\|PTGER4\|RC3H1\|RAB27A\|SLC11A1\|LCP1\|RPS6\|PSEN1\|FCER1G\|RORA\|IL6 |
|  | GO_POSITIVE_REGULATION_OF_CYTOKINE_BIOSYNTHETIC_PROCESS | 25 | HMOX1\|THBS1\|TICAM1\|TNFRSF8\|HSPB1\|BCL3\|CCL20\|MAPKAPK2\|RELA\|ZFPM1\|GLMN\|EREG\|IRF1\|LTB\|CD80\|BCL10\|CD276\|CEBPG\|IL1B\|TNF\|TRAF6\|SYK\|PTAFR\|STAT5B\|TBK1 |
|  | GO_CHEMOKINE_RECEPTOR_BINDING | 25 | CXCL14\|CXCL16\|CCL28\|CCL5\|CCL20\|CCL26\|CCL3\|C5\|CCL2\|STAT3\|CXCL12\|CXCL5\|S100A14\|CXCL1\|CCL4\|CCL22\|IL8\|CNIH4\|ITCH\|YARS\|JAK1\|CCRL2\|CXCL2\|CREB3\|CXCL3 |
|  | GO_REGULATION_OF_GRANULOCYTE_CHEMOTAXIS | 23 | CAMK1D\|CCL2\|DAPK2\|MPP1\|THBS1\|IL1B\|SLIT2\|TRPV4\|RARRES2\|EDNRA\|S100A14\|RAC1\|CXCL1\|C5AR1\|C3AR1\|IL23A\|IL8\|CCL5\|CD74\|CSF1\|CXCL2\|CXCL3\|CCR7 |
|  | GO_CHEMOKINE_MEDIATED_SIGNALING_PATHWAY | 21 | CCR1\|CCL2\|PTK2B\|CXCL12\|CXCL5\|CXCL1\|CCL4\|CCL22\|IL8\|CCL5\|CCL20\|CCR3\|XCR1\|CXCR4\|CCRL2\|CXCL2\|CIB1\|CXCL3\|CCL26\|CCL3\|CCR7 |
|  | GO_CD4_POSITIVE_ALPHA_BETA_T_CELL_ACTIVATION | 21 | HMGB1\|PTGER4\|RC3H1\|SEMA4A\|CEBPB\|ATP7A\|GATA3\|BCL3\|RORA\|LEF1\|STAT6\|RORC\|BATF\|FOXP3\|ZFPM1\|BRAF\|IL6\|RELB\|FOXP1\|STOML2\|RC3H2 |
|  | GO_T_CELL_DIFFERENTIATION_INVOLVED_IN_IMMUNE_RESPONSE | 20 | EOMES\|HMGB1\|PTGER4\|RC3H1\|SEMA4A\|FCER1G\|ATP7A\|GATA3\|BCL3\|LEF1\|RORA\|STAT6\|RORC\|BATF\|ZFPM1\|RELB\|IL6\|FOXP1\|RC3H2\|TSC1 |
|  | GO_CHEMOKINE_ACTIVITY | 17 | C5\|CCL2\|CXCL14\|CXCL16\|CXCL12\|CXCL5\|CCL28\|CXCL1\|CCL4\|CCL22\|IL8\|CCL5\|CCL20\|CXCL2\|CCL26\|CXCL3\|CCL3 |
|  | GO_ANTIMICROBIAL_HUMORAL_RESPONSE | 17 | HLA-E\|RPL39\|SPON2\|IGHA1\|HIST1H2BG\|B2M\|HIST1H2BJ\|HIST1H2BK\|HIST1H2BI\|HLA-A\|HIST1H2BF\|HIST2H2BE\|HIST1H2BC\|BCL3\|PLA2G6\|ADM\|APP |
| Response to Starvation | GO_CELLULAR_RESPONSE_TO_EXTERNAL_STIMULUS | 185 | PPM1D\|BMPR2\|DEPDC5\|CDKN1A\|HFE\|GLUL\|WDR59\|MAX\|RRAGB\|AKR1C3\|SRD5A1\|NPRL3\|WRN\|EIF2AK4\|MAP1LC3B\|RALB\|ZFYVE1\|FOSL1\|WNT4\|FBXO22\|GCN1L1\|PRKAA1\|SLC38A2\|COL1A1\|RNF152\|RRAGC\|BNIP3\|GLRX2\|UCP2\|CAV1\|WDR24\|FADD\|ATG7\|USF1\|USP33\|RRAGA\|IL1B\|KIAA1324\|SEH1L\|ADORA2B\|HSPA5\|ADNP\|MAG\|CYP24A1\|CASP8AP2\|ITGB1\|NCOA1\|MDM2\|CRADD\|KCNJ4\|CDKN2B\|IGF2\|SIPA1\|LTBR\|XBP1\|CCNE1\|AKT1\|HABP4\|TNFRSF10A\|NR4A2\|BAK1\|ATP2B1\|FZD1\|PIM1\|BAG3\|BRIP1\|EIF2S1\|HMOX1\|KIF26A\|GAS6\|TXN2\|USF2\|SLC9A1\|ATP1A1\|FOS\|EIF2AK2\|ITGA6\|CHMP1A\|ITGA2\|EIF2AK3\|CBS\|VDR\|MAP2K4\|EDNRA\|PDK2\|RAC1\|MAP3K2\|PRDM4\|MTPN\|NPPA\|TNFRSF8\|RPTOR\|NUDT1\|ATF4\|CNN2\|PPP1R9B\|SKP2\|HDAC7\|ASNS\|SRF\|HSPA8\|BHLHA15\|NFE2L2\|TBL2\|MYD88\|MTOR\|PPP1R15A\|GSDMD\|SPHK1\|FOLR1\|ATF3\|HDAC4\|PMAIP1\|PIK3C3\|PIK3R4\|ATG14\|RRP8\|MAPK8\|ZC3H12A\|BCL2\|UPP1\|MTMR3\|IMPACT\|BMP6\|VLDLR\|SLC2A1\|PICK1\|BECN1\|SH3GLB1\|MYBBP1A\|NUAK2\|CPEB4\|TP53\|BMP4\|CCL5\|ICAM1\|SOX9\|IRF1\|TSC1\|LYN\|PAWR\|NFKB1\|ANKRD1\|GADD45A\|PTGER4\|INHBB\|SIRT1\|FAS\|GSK3B\|CTSB\|MAP3K1\|CASP8\|CTNNB1\|CASP2\|AIFM1\|BCL10\|SLC39A4\|COMT\|NPRL2\|KLF10\|DNAJC15\|TLR5\|SIRT2\|FADS1\|ATG5\|TNFRSF10B\|GAS2L1\|DSC2\|PPARG\|FOXO1\|ULK1\|JUN\|CADPS2\|GBA\|GABARAPL1\|SREBF1\|RRAGD\|PDK4\|SREBF2\|MIOS\|TNFRSF1A\|DAP\|GABARAPL2\|MAP3K14\|EHMT2 |
|  | GO_RESPONSE_TO_STARVATION | 111 | PPM1D\|BMPR2\|DEPDC5\|CDKN1A\|SLC18A2\|HFE\|GLUL\|WDR59\|MAX\|RRAGB\|AKR1C3\|SRD5A1\|ZFP36\|NPRL3\|ADSSL1\|WRN\|EIF2AK4\|BDH1\|MAP1LC3B\|RALB\|ZFYVE1\|HDDC3\|WNT4\|FBXO22\|PRKAA1\|GCN1L1\|SLC38A2\|RNF152\|RRAGC\|TBC1D5\|UCP2\|CAV1\|WDR24\|ATG7\|AACS\|USP33\|RRAGA\|KIAA1324\|SEH1L\|HSPA5\|XBP1\|EIF2S1\|GAS6\|EIF2AK2\|EIF2AK3\|ATF4\|ASNS\|HSPA8\|BHLHA15\|NFE2L2\|TBL2\|PPP1R15A\|SPHK1\|DDIT3\|ATF3\|PMAIP1\|PIK3C3\|PIK3R4\|ATG14\|RRP8\|ZC3H12A\|BCL2\|UPP1\|MTMR3\|IMPACT\|VLDLR\|SLC2A1\|PICK1\|BECN1\|SH3GLB1\|MYBBP1A\|NUAK2\|CPEB4\|TP53\|INHBB\|SIRT1\|SLC39A4\|COMT\|NPRL2\|KLF10\|DNAJC15\|ACADS\|HMGCL\|SIRT2\|FADS1\|DHODH\|ATG5\|GAS2L1\|DSC2\|PPARG\|BCAS3\|GNPAT\|ULK2\|FOXO1\|ULK1\|JUN\|CADPS2\|GBA\|GABARAPL1\|SREBF1\|RRAGD\|PDK4\|SREBF2\|LRP11\|MIOS\|DAP\|OXCT1\|ADM\|GABARAPL2\|ACAT1\|EHMT2 |
|  | GO_CELLULAR_RESPONSE_TO_STARVATION | 91 | PPM1D\|PIK3R4\|BMPR2\|ATG14\|DEPDC5\|RRP8\|CDKN1A\|ZC3H12A\|BCL2\|HFE\|UPP1\|MTMR3\|GLUL\|IMPACT\|WDR59\|VLDLR\|MAX\|SLC2A1\|RRAGB\|PICK1\|AKR1C3\|BECN1\|SRD5A1\|SH3GLB1\|MYBBP1A\|NPRL3\|NUAK2\|CPEB4\|WRN\|EIF2AK4\|TP53\|MAP1LC3B\|RALB\|ZFYVE1\|WNT4\|FBXO22\|PRKAA1\|GCN1L1\|SLC38A2\|RNF152\|RRAGC\|UCP2\|CAV1\|WDR24\|ATG7\|USP33\|RRAGA\|KIAA1324\|SEH1L\|INHBB\|HSPA5\|SIRT1\|XBP1\|EIF2S1\|GAS6\|EIF2AK2\|EIF2AK3\|ATF4\|ASNS\|HSPA8\|BHLHA15\|SLC39A4\|COMT\|NPRL2\|NFE2L2\|KLF10\|DNAJC15\|TBL2\|SIRT2\|FADS1\|ATG5\|PPP1R15A\|GAS2L1\|DSC2\|SPHK1\|FOXO1\|JUN\|CADPS2\|GBA\|ATF3\|GABARAPL1\|SREBF1\|RRAGD\|PDK4\|SREBF2\|MIOS\|DAP\|GABARAPL2\|PMAIP1\|EHMT2\|PIK3C3 |
|  | GO_MONOSACCHARIDE_TRANSPORT | 34 | SESN2\|HK2\|SLC2A13\|STXBP3\|EZR\|FABP5\|KLF15\|YES1\|SLC23A1\|SORBS1\|SLC2A1\|EDNRA\|G6PC3\|DRD1\|BRAF\|PPARD\|TSC1\|EDN1\|VIL1\|HK1\|PLS1\|SLC2A10\|M6PR\|SLC2A4\|SLC26A5\|SLC2A6\|SLC2A12\|AKT1\|STXBP4\|SLC23A2\|SLC37A4\|SORT1\|HNF1A\|SLC2A8 |
|  | GO_NUCLEOTIDE_SUGAR_METABOLIC_PROCESS | 30 | GNPNAT1\|SLC35A3\|UGDH\|MGAT1\|FUT8\|CSGALNACT1\|GMDS\|RENBP\|GUK1\|PGM3\|GALT\|UXS1\|GMPPB\|GNE\|UGP2\|TGDS\|PMM1\|UGGT2\|UAP1L1\|GFPT1\|NAGK\|SLC35D1\|MPI\|UGGT1\|DPM1\|TSTA3\|GFPT2\|EXTL2\|UAP1\|DPAGT1 |
|  | GO_CELLULAR_RESPONSE_TO_GLUCOSE_STARVATION | 28 | PIK3R4\|ATG14\|RRP8\|ZC3H12A\|BCL2\|UPP1\|MTMR3\|EIF2AK3\|IMPACT\|VLDLR\|SLC2A1\|PICK1\|BECN1\|SH3GLB1\|MYBBP1A\|NUAK2\|CPEB4\|ATF4\|TP53\|ASNS\|PRKAA1\|BHLHA15\|NFE2L2\|TBL2\|HSPA5\|XBP1\|PMAIP1\|PIK3C3 |
|  | GO_GLUTAMATE_RECEPTOR_BINDING | 26 | FUS\|SHANK2\|DNM3\|GNAS\|HOMER1\|CTNNB1\|FLOT2\|OPHN1\|IL1R1\|CALM1\|FLOT1\|GRIN1\|NETO2\|CANX\|DLG4\|CALM3\|DLG1\|SHANK3\|ESR1\|SYNDIG1\|DLG2\|DLG3\|RAB4A\|HOMER2\|GSK3B\|DRD2 |
| ER Stress | GO_RESPONSE_TO_ENDOPLASMIC_RETICULUM_STRESS | 191 | RNF185\|FBXO6\|ALOX15\|THBS1\|SRPX\|USP13\|TMEM67\|MAP3K5\|SELK\|UFC1\|EEF2\|TMBIM6\|ATP2A1\|ITPR1\|STT3B\|SEL1L\|EIF2AK4\|OS9\|TXNDC11\|HM13\|UBE2J2\|FBXO2\|FLOT1\|PDIA4\|RNF139\|UBE2G2\|NRBF2\|PSMC6\|PIK3R2\|VAPB\|EP300\|CREB3L2\|ERN1\|LMNA\|ANKZF1\|DNAJB9\|HERPUD1\|PREB\|TATDN2\|HSPA5\|ATXN3\|CALR\|ARFGAP1\|ERO1L\|ATP6V0D1\|IL8\|ACADVL\|EDEM1\|CTH\|SRPR\|HSP90B1\|WFS1\|STC2\|CCND1\|VCP\|XBP1\|DNAJB11\|SEC63\|BAK1\|SRPRB\|UFD1L\|STUB1\|EIF2S1\|KLHDC3\|AMFR\|DAB2IP\|PARP16\|EIF2AK2\|DERL2\|EIF2AK3\|HDGF\|DERL1\|TLN1\|BAX\|FKBP14\|SDF2L1\|DNAJC3\|PPP2R5B\|ATF4\|RNF5\|CXXC1\|SERP1\|ASNS\|DDX11\|GFPT1\|HYOU1\|PTPN1\|MBTPS2\|TPP1\|WIPI1\|BHLHA15\|CUL7\|ADD1\|EDEM3\|NFE2L2\|CCL2\|CREB3L1\|ATF6\|TBL2\|PDIA6\|RHBDD1\|PPP1R15A\|ASNA1\|TOR1A\|MBTPS1\|C19ORF10\|ZBTB17\|TSPYL2\|DDIT3\|SEC61A2\|ATF3\|CTDSP2\|AARS\|PARK2\|YIF1A\|SEC61G\|CREB3\|SEC61A1\|SEC61B\|SEC62\|EXTL3\|PMAIP1\|BCL2\|TP53\|CEBPB\|PPP1R15B\|GSK3B\|ATG10\|ERLIN2\|ATP2A2\|TTC23L\|PPP2CB\|UBQLN1\|PSMC2\|UBE2J1\|UBE2K\|UBXN8\|TRIM25\|FOXRED2\|USP19\|TMEM129\|BBC3\|UFM1\|DNAJB2\|CASP4\|COL4A3BP\|PSMC4\|TRAF2\|DNAJC10\|UBA5\|UBXN4\|PSMC1\|TRIM13\|MAN1B1\|AUP1\|BRSK2\|AIFM1\|ERLEC1\|TMEM33\|TMX4\|APAF1\|PDIA3\|BCL2L11\|FAF2\|NCK2\|TMUB1\|SCAMP5\|TNFRSF10B\|TMX3\|UFL1\|JKAMP\|CHAC1\|MARCH6\|UBE4B\|PML\|JUN\|ERP44\|P4HB\|PLA2G6\|DDRGK1\|BAG6\|TARDBP\|TMX1\|PIK3R1\|ERP27\|PSMC3\|TRIB3\|UBQLN2\|KIAA0368\|AGR2\|NPLOC4 |
|  | GO_RESPONSE_TO_TOPOLOGICALLY_INCORRECT_PROTEIN | 128 | FBXO6\|THBS1\|TMBIM6\|STT3B\|UBE2J2\|VAPB\|EP300\|CREB3L2\|ERN1\|LMNA\|ANKZF1\|DNAJB9\|HERPUD1\|PREB\|TATDN2\|HSPA5\|ATXN3\|CALR\|ARFGAP1\|ERO1L\|ATP6V0D1\|IL8\|ACADVL\|EDEM1\|CTH\|SRPR\|HSP90B1\|WFS1\|STC2\|CCND1\|VCP\|XBP1\|DNAJB11\|SEC63\|BAK1\|SRPRB\|UFD1L\|STUB1\|EIF2S1\|KLHDC3\|AMFR\|DAB2IP\|PARP16\|EIF2AK2\|DERL2\|EIF2AK3\|HDGF\|DERL1\|TLN1\|BAX\|FKBP14\|SDF2L1\|HSPA2\|DNAJC3\|HSPB1\|PPP2R5B\|HSP90AB1\|ATF4\|RNF5\|MANF\|CXXC1\|HSPD1\|CLU\|SERP1\|TOR1B\|ASNS\|DNAJB1\|RNF126\|HSPA1L\|DDX11\|HSPA8\|GFPT1\|HSPA4\|HYOU1\|DNAJB4\|PTPN1\|SERPINH1\|MBTPS2\|HSP90AA1\|TPP1\|WIPI1\|MFN2\|BHLHA15\|DNAJA1\|CUL7\|F12\|ADD1\|HSPH1\|EDEM3\|DNAJB5\|NFE2L2\|HERPUD2\|CCL2\|HSPA4L\|CREB3L1\|ATF6\|TBL2\|HDAC6\|PDIA6\|RHBDD1\|PPP1R15A\|ASNA1\|TOR1A\|MBTPS1\|PACRG\|C19ORF10\|ZBTB17\|TSPYL2\|DDIT3\|SEC61A2\|ATF3\|CTDSP2\|AARS\|PARK2\|YIF1A\|SEC61G\|CREB3\|SEC61A1\|SEC61B\|SEC62\|EXTL3\|TMEM129\|DNAJB2\|UBXN4\|FAF2\|JKAMP\|CHAC1\|ERP44 |
|  | GO_CELLULAR_RESPONSE_TO_TOPOLOGICALLY_INCORRECT_PROTEIN | 95 | VAPB\|EP300\|CREB3L2\|ERN1\|LMNA\|ANKZF1\|DNAJB9\|HERPUD1\|PREB\|TATDN2\|HSPA5\|ATXN3\|CALR\|ARFGAP1\|ERO1L\|ATP6V0D1\|IL8\|ACADVL\|EDEM1\|CTH\|SRPR\|HSP90B1\|WFS1\|STC2\|CCND1\|VCP\|XBP1\|DNAJB11\|SEC63\|BAK1\|SRPRB\|UFD1L\|STUB1\|EIF2S1\|KLHDC3\|AMFR\|DAB2IP\|PARP16\|EIF2AK2\|DERL2\|EIF2AK3\|HDGF\|DERL1\|TLN1\|BAX\|FKBP14\|SDF2L1\|DNAJC3\|PPP2R5B\|ATF4\|RNF5\|CXXC1\|SERP1\|ASNS\|RNF126\|DDX11\|GFPT1\|HYOU1\|PTPN1\|MBTPS2\|TPP1\|WIPI1\|BHLHA15\|CUL7\|ADD1\|EDEM3\|NFE2L2\|CCL2\|CREB3L1\|ATF6\|TBL2\|HDAC6\|PDIA6\|RHBDD1\|PPP1R15A\|ASNA1\|TOR1A\|MBTPS1\|PACRG\|C19ORF10\|ZBTB17\|TSPYL2\|DDIT3\|SEC61A2\|ATF3\|CTDSP2\|AARS\|PARK2\|YIF1A\|SEC61G\|CREB3\|SEC61A1\|SEC61B\|SEC62\|EXTL3 |
|  | GO_RESPONSE_TO_AMINO_ACID | 78 | RRAGB\|EEF2\|TMBIM6\|NRF1\|CPEB4\|CCL5\|ICAM1\|CASP3\|RELA\|SOCS1\|COL1A1\|RRAGC\|RRAGA\|LYN\|EDN1\|PTGER4\|TNF\|CEBPB\|XBP1\|FAS\|HMBS\|IL6\|GSK3B\|CDKN1B\|HNRNPK\|CAPN2\|ASCL1\|BCL2L1\|ZNF354A\|RPTOR\|DNMT3A\|ABCG2\|OGG1\|LAMTOR1\|ZEB1\|DRD1\|ASNS\|COL6A1\|UBR2\|MGMT\|TYMS\|GLRB\|COL16A1\|ASS1\|CTGF\|EGFR\|PDGFC\|AIFM1\|NEURL\|UBR1\|GSS\|CREB1\|NAIP\|SLC38A9\|CCL2\|GLRA3\|HNRNPD\|COL3A1\|COL5A2\|TIMP3\|MTOR\|IPO5\|LAMTOR2\|UFL1\|GSN\|COL4A1\|SH3BP4\|PEMT\|FOLR1\|LAMTOR3\|RAPGEF3\|MTHFR\|CPEB3\|RRAGD\|AARS\|CPEB1\|PIK3C3\|ALAD |
|  | GO_GOLGI_ORGANIZATION | 76 | UBXN2A\|GAK\|GOLGA2\|TMED5\|TMED2\|SYNE1\|GBF1\|BAG5\|ATL2\|OPTN\|LMAN1\|TBC1D20\|FBXW8\|COG7\|COG4\|CSNK1D\|YWHAZ\|ATL3\|PRKD1\|ARFGEF1\|CDK1\|HACE1\|SEC23IP\|DYNC2H1\|ARHGAP21\|OBSL1\|RAB2A\|GCC2\|GOLGA5\|ARL1\|CDC42\|SURF4\|GOLPH3\|RAB7L1\|UBXN2B\|TJAP1\|HTT\|TMED10\|BHLHA15\|USP6NL\|CUL7\|VTI1A\|VRK1\|CSNK1A1\|GORASP1\|RAB1A\|COG1\|PRMT5\|GOLPH3L\|PLEKHM2\|VMP1\|DNAJC28\|NSFL1C\|COG2\|BCAS3\|TMED9\|CLASP1\|ATP8B2\|DYM\|ZW10\|ARHGEF7\|VCPIP1\|ATP8B1\|GOLGB1\|ATP8B3\|STK25\|COG3\|CLASP2\|PDCD10\|MYO18A\|KIFC3\|BLZF1\|PLK3\|STX17\|NPLOC4\|VAMP4 |
|  | GO_IRE1_MEDIATED_UNFOLDED_PROTEIN_RESPONSE | 45 | KLHDC3\|HDGF\|TLN1\|FKBP14\|DNAJC3\|PPP2R5B\|CXXC1\|SERP1\|DDX11\|GFPT1\|HYOU1\|PTPN1\|TPP1\|WIPI1\|CUL7\|ADD1\|ERN1\|LMNA\|DNAJB9\|PDIA6\|PREB\|TATDN2\|HSPA5\|ASNA1\|ARFGAP1\|C19ORF10\|ATP6V0D1\|ZBTB17\|TSPYL2\|ACADVL\|EDEM1\|SEC61A2\|SRPR\|CTDSP2\|WFS1\|YIF1A\|SEC61G\|XBP1\|DNAJB11\|SEC61A1\|SEC63\|SEC61B\|SEC62\|SRPRB\|EXTL3 |
|  | GO_DEMETHYLATION | 41 | CYP1A1\|KDM1A\|KDM6B\|KDM5C\|KDM3A\|KDM1B\|KDM4B\|ALKBH3\|PHF8\|CYP51A1\|KDM4D\|KDM4A\|JHDM1D\|CYP1A2\|ARID5B\|TET2\|CYP2D6\|CYP3A5\|PPME1\|TDG\|POR\|APEX1\|ALKBH1\|CYP2C8\|TET3\|UBE2B\|TET1\|JMJD6\|KDM3B\|FTO\|JMJD1C\|ALKBH2\|KDM2B\|KDM5A\|KDM5B\|KDM2A\|PHF2\|KDM6A\|ALKBH5\|KDM4C\|ALKBH4 |
|  | GO_INTRINSIC_APOPTOTIC_SIGNALING_PATHWAY_IN_RESPONSE_TO_ENDOPLASMIC_RETICULUM_STRESS | 31 | DAB2IP\|BCL2\|MAP3K5\|SELK\|UBE2K\|BAX\|TMBIM6\|ATP2A1\|ITPR1\|BBC3\|ATF4\|CASP4\|TRAF2\|DNAJC10\|BRSK2\|AIFM1\|APAF1\|ERN1\|TNFRSF10B\|PPP1R15A\|CHAC1\|ERO1L\|CEBPB\|PML\|DDIT3\|BAG6\|XBP1\|TRIB3\|BAK1\|GSK3B\|PMAIP1 |
|  | GO_DEMETHYLASE_ACTIVITY | 30 | CYP1A1\|KDM1A\|KDM6B\|JARID2\|KDM5C\|KDM3A\|KDM1B\|KDM4B\|ALKBH3\|PHF8\|CYP51A1\|KDM4D\|KDM4A\|JHDM1D\|CYP1A2\|ARID5B\|JMJD6\|KDM3B\|FTO\|JMJD1C\|ALKBH2\|KDM2B\|KDM5A\|KDM5B\|KDM2A\|PHF2\|KDM6A\|ALKBH5\|KDM4C\|ALKBH4 |
|  | GO_ER_NUCLEUS_SIGNALING_PATHWAY | 29 | EIF2S1\|EIF2A\|ATP2A2\|SCAP\|EIF2AK3\|INSIG1\|ATF4\|TP53\|ASNS\|MBTPS2\|INSIG2\|NFE2L2\|CCL2\|LMNA\|ATF6\|HERPUD1\|HSPA5\|CALR\|MBTPS1\|IL8\|DDIT3\|ATF3\|HSP90B1\|WFS1\|XBP1\|PPP1R15B\|GSK3B\|ATG10\|ERLIN2 |
|  | GO_NEGATIVE_REGULATION_OF_RESPONSE_TO_ENDOPLASMIC_RETICULUM_STRESS | 28 | DERL2\|UBE2J1\|TMBIM6\|DNAJC3\|OS9\|HYOU1\|PTPN1\|UBE2G2\|ERLEC1\|CREB3L1\|NCK2\|HERPUD1\|PPP1R15A\|OPA1\|PARK7\|GRINA\|GNB2L1\|USP14\|WFS1\|USP25\|BFAR\|PARK2\|SVIP\|UBAC2\|CREB3\|XBP1\|UBXN1\|PPP1R15B |
|  | GO_REGULATION_OF_TYROSINE_PHOSPHORYLATION_OF_STAT3_PROTEIN | 26 | IL18\|PPP2R1A\|INPP5F\|CTF1\|NF2\|VEGFA\|SOCS1\|CRLF1\|LIF\|STAT3\|PTK6\|CSF1R\|PTGER4\|HDAC2\|JAK2\|ARL2BP\|IL6ST\|HES1\|SOCS3\|STAP2\|GHR\|IL23A\|FGFR3\|PTPN2\|IL6R\|IL6 |
|  | GO_PROTEIN_DEALKYLATION | 24 | KDM1A\|PPME1\|KDM6B\|KDM5C\|KDM3A\|KDM1B\|KDM4B\|UBE2B\|PHF8\|KDM4D\|KDM4A\|JHDM1D\|ARID5B\|JMJD6\|KDM3B\|JMJD1C\|KDM2B\|KDM5A\|KDM5B\|KDM2A\|PHF2\|KDM6A\|KDM4C\|ALKBH4 |
|  | GO_REGULATION_OF_ENDOPLASMIC_RETICULUM_UNFOLDED_PROTEIN_RESPONSE | 24 | ERN1\|DAB2IP\|BCL2L11\|NCK2\|PPP1R15A\|HSPA5\|BAX\|TMBIM6\|POMT2\|SDF2L1\|POMT1\|BBC3\|SDF2\|PTPN2\|GNB2L1\|WFS1\|BFAR\|PIK3R1\|PTPN1\|XBP1\|PPP1R15B\|BAK1\|AGR2\|TMEM33 |
|  | GO_PROTEIN_LOCALIZATION_TO_GOLGI_APPARATUS | 23 | OPTN\|NRAS\|VPS13A\|COG7\|CSNK1D\|IFT20\|PAQR3\|ATP9B\|GBF1\|GCC1\|PACS1\|SORL1\|ARFRP1\|OBSL1\|GCC2\|ARL1\|VPS13D\|GOLPH3\|VPS13C\|GOLGA4\|RAB6A\|TRIP11\|RAB33B |
|  | GO_HISTONE_DEMETHYLASE_ACTIVITY | 22 | KDM1A\|KDM6B\|JARID2\|KDM5C\|KDM3A\|KDM1B\|KDM4B\|PHF8\|KDM4D\|KDM4A\|JHDM1D\|ARID5B\|JMJD6\|KDM3B\|JMJD1C\|KDM2B\|KDM5A\|KDM5B\|KDM2A\|PHF2\|KDM6A\|KDM4C |
|  | GO_REGULATION_OF_ENDOPLASMIC_RETICULUM_STRESS_INDUCED_INTRINSIC_APOPTOTIC_SIGNALING_PATHWAY | 22 | CREB3L1\|BCL2L11\|NCK2\|HERPUD1\|EIF2AK3\|OPA1\|TMBIM6\|SIRT1\|PARK7\|BBC3\|SERINC3\|ERP29\|GRINA\|DDIT3\|PTPN2\|WFS1\|PARK2\|HYOU1\|CREB3\|PTPN1\|XBP1\|PMAIP1 |
|  | GO_POSITIVE_REGULATION_OF_TYROSINE_PHOSPHORYLATION_OF_STAT3_PROTEIN | 21 | IL18\|LIF\|STAT3\|PTK6\|CTF1\|CSF1R\|HDAC2\|PTGER4\|JAK2\|ARL2BP\|IL6ST\|VEGFA\|HES1\|SOCS3\|STAP2\|IL23A\|GHR\|FGFR3\|IL6R\|IL6\|CRLF1 |
|  | GO_POSITIVE_REGULATION_OF_TRANSCRIPTION_FROM_RNA_POLYMERASE_II_PROMOTER_IN_RESPONSE_TO_STRESS | 18 | NFE2L2\|CREB3L1\|ATF6\|MAPK7\|HSPA5\|NOTCH1\|VEGFA\|CEBPB\|TP53\|RBPJ\|ATF4\|DDIT3\|KLF2\|ATF3\|GCN1L1\|XBP1\|MBTPS2\|CHD6 |
|  | GO_PROTEIN_EXIT_FROM_ENDOPLASMIC_RETICULUM | 18 | FAF2\|DERL2\|HERPUD1\|RANGRF\|RHBDD1\|LMAN1\|DERL1\|TMED9\|TMEM129\|SEL1L\|HM13\|HSP90B1\|SURF4\|VCP\|SEC61B\|AUP1\|UFD1L\|NPLOC4 |
|  | GO_ENDOPLASMIC_RETICULUM_TO_CYTOSOL_TRANSPORT | 17 | FAF2\|DERL2\|HERPUD1\|RHBDD1\|DERL1\|NOL3\|TMEM129\|SEL1L\|HM13\|HSP90B1\|VCP\|SEC61B\|AUP1\|CCL3\|UFD1L\|FKBP1B\|NPLOC4 |
| Sterol Biosynthesis | GO_ALCOHOL_BIOSYNTHETIC_PROCESS | 74 | DHCR7\|CYB5R3\|HMGCS1\|MVK\|MVD\|ACLY\|INSIG1\|HMGCR\|PMVK\|AKR1C3\|CYP51A1\|HSD17B7\|IDI2\|MSMO1\|LBR\|NSDHL\|CYB5R1\|SQLE\|FDFT1\|PRKAG2\|AKR1B1\|GGPS1\|G6PD\|EBP\|PRKAA1\|ARV1\|FDPS\|INSIG2\|C14ORF1\|DHCR24\|CNBP\|IDI1\|DPAGT1\|NUS1\|PARK7\|IMPA1\|SAMD8\|PTAFR\|ACHE\|PLCG2\|SLC44A1\|SGMS1\|SCP2\|CHKA\|SGMS2\|IPMK\|LPIN2\|GOT1\|FABP5\|AGPAT6\|IMPA2\|SLC44A3\|LPIN1\|ACER2\|SPTLC2\|LPCAT2\|ABHD3\|SPHK2\|CACNA1H\|CEPT1\|SLC27A1\|CDS1\|CYP2R1\|ISYNA1\|CHPT1\|ACER3\|SLC44A2\|IMPAD1\|LPIN3\|SPTLC1\|PHOSPHO1\|SPHK1\|PEMT\|GBA |
|  | GO_STEROL_METABOLIC_PROCESS | 71 | CEL\|CEBPA\|DHCR7\|ABCG1\|CYB5R3\|SNX17\|HMGCS1\|LRP5\|MVK\|CYP19A1\|MVD\|OSBPL5\|ACLY\|HDLBP\|INSIG1\|CYP46A1\|HMGCR\|VLDLR\|SOAT1\|PMVK\|SCARB1\|CYP51A1\|LDLRAP1\|HSD17B7\|ABCA1\|IDI2\|CUBN\|MSMO1\|CAT\|LBR\|NSDHL\|PTCHD2\|CYB5R1\|NPC2\|SQLE\|CYP4V2\|STARD3\|FDFT1\|RXRA\|PRKAG2\|NPC1\|GGPS1\|G6PD\|LIPA\|EBP\|FDX1\|PRKAA1\|APOL2\|ARV1\|CLN8\|FDPS\|FDXR\|INSIG2\|PPARD\|C14ORF1\|APOBR\|DHCR24\|APOE\|CNBP\|LDLR\|IDI1\|APP\|ERLIN2\|SCAP\|SORL1\|MBTPS2\|MBTPS1\|SREBF1\|SREBF2\|TRERF1\|EBPL |
|  | GO_STEROID_BIOSYNTHETIC_PROCESS | 67 | HSD17B6\|DHCR7\|HSD17B12\|CYB5R3\|ACOX2\|HMGCS1\|HSD17B11\|MVK\|HINT2\|MVD\|CYP19A1\|SRD5A3\|ACLY\|INSIG1\|HMGCR\|CYP46A1\|PMVK\|AKR1C3\|CYP51A1\|SCARB1\|SRD5A1\|HSD17B7\|IDI2\|MSMO1\|LBR\|NSDHL\|CYB5R1\|SQLE\|FDFT1\|STARD3\|PRKAG2\|AKR1B1\|GGPS1\|G6PD\|WNT4\|FDX1\|EBP\|PRKAA1\|ARV1\|FDXR\|FDPS\|INSIG2\|C14ORF1\|DHCR24\|CNBP\|IDI1\|TSPO\|SCP2\|CACNA1H\|CYP2R1\|SLC27A2\|HSD3B7\|SDR42E1\|PRLR\|STARD5\|ACBD3\|HSD11B2\|MED1\|HSD17B14\|SLC27A5\|HSD17B8\|PBX1\|ADM\|ACOT8\|HSD17B4\|TRERF1\|AKR1B15 |
|  | GO_STEROL_BIOSYNTHETIC_PROCESS | 30 | DHCR7\|CYB5R3\|HMGCS1\|MVK\|MVD\|ACLY\|INSIG1\|HMGCR\|PMVK\|CYP51A1\|HSD17B7\|IDI2\|MSMO1\|LBR\|NSDHL\|CYB5R1\|SQLE\|FDFT1\|PRKAG2\|GGPS1\|G6PD\|EBP\|PRKAA1\|ARV1\|FDPS\|INSIG2\|C14ORF1\|DHCR24\|CNBP\|IDI1 |
|  | GO_ISOPRENOID_BIOSYNTHETIC_PROCESS | 19 | CYP1A1\|HMGCS1\|MVK\|PDSS1\|MVD\|BCMO1\|DPAGT1\|ISPD\|HMGCR\|NUS1\|PMVK\|COQ2\|RDH10\|IDI2\|PDSS2\|FDFT1\|GGPS1\|FDPS\|IDI1 |
| Stress Response | GO_RESPONSE_TO_HYDROGEN_PEROXIDE | 81 | BCL2\|MAP3K5\|IMPACT\|EEF2\|AREG\|CAT\|DUSP1\|PPP5C\|AKR1B1\|ADAM9\|FOSL1\|RPS3\|MB\|CASP3\|NR4A3\|PRKAA1\|HBB\|RELA\|COL1A1\|BNIP3\|PCGF2\|GLRX2\|KLF6\|STK24\|FKBP1B\|NET1\|EP300\|LDHA\|ECT2\|KPNA4\|KLF4\|MAPK7\|EZH2\|TXNIP\|HDAC2\|APTX\|MST4\|ADA\|ZNF277\|SIRT1\|AGER\|PRKCD\|PARK7\|SOD1\|PLEKHA1\|F3\|CBX8\|SETX\|RHOB\|KLF2\|FXN\|PPIF\|MDM2\|FAS\|IL6\|PPP1R15B\|BAK1\|GSK3B\|PRDX3\|HMOX1\|KDM6B\|SLC8A1\|PPP2CB\|PTK2B\|CDK1\|STAT6\|TNFAIP3\|AIFM1\|NFE2L2\|APEX1\|HDAC6\|SDC1\|SPHK1\|FOXO1\|ANXA1\|JUN\|DDIT3\|SRC\|STK25\|PDCD10\|ABL1 |
|  | GO_PATTERN_RECOGNITION_RECEPTOR_SIGNALING_PATHWAY | 73 | PIK3R4\|IRAK1\|RELA\|IRAK2\|FADD\|IKBKB\|TNIP2\|S100A14\|TRAF6\|ITCH\|HSP90B1\|UBE2D3\|FFAR2\|SCARA3\|IRAK4\|BIRC3\|IRF3\|UNC93B1\|DDX58\|MAVS\|COLEC12\|IKBKG\|TBK1\|UBE2D1\|FAM105B\|UBE2D2\|CTSB\|TAB2\|TAB3\|RIPK1\|MAP3K1\|CTSK\|UBB\|NOD1\|TICAM1\|CASP8\|UBE2N\|RFTN1\|NOD2\|PRKCE\|CLEC7A\|HSPD1\|CTSS\|MAP2K6\|BIRC2\|LGMN\|TNFAIP3\|TAB1\|MAPKAPK2\|IKBKE\|UBC\|ITGAM\|RPS27A\|BCL10\|TRAF3\|IFIH1\|UBA52\|RPS6KA3\|LSM14A\|TANK\|TLR5\|TRIM5\|MYD88\|MAP3K7\|CNPY3\|TNIP1\|IRF7\|MAPKAPK3\|CYLD\|TLR2\|RIPK2\|NFKBIA\|PIK3C3 |
|  | GO_HOMOPHILIC_CELL_ADHESION_VIA_PLASMA_MEMBRANE_ADHESION_MOLECULES | 64 | PVRL1\|PCDHB4\|RET\|PCDHB3\|CDH24\|PCDHB2\|PCDHB13\|PCDHB10\|PCDHB14\|MPZL2\|PCDHB16\|PCDHB11\|PCDHB9\|NPTN\|CDH3\|CDHR3\|DSCAM\|CDH2\|CDH18\|CDH12\|CDH1\|PCDH19\|PCDHA4\|PCDHB12\|CELSR2\|CDH26\|PCDHGA1\|CADM1\|PCDH10\|CELSR1\|PTPRT\|SDK2\|AMIGO1\|ITGB1\|DSCAML1\|PVRL2\|AMIGO2\|IGSF9B\|SMAGP\|SDK1\|PVRL4\|PCDH8\|CELSR3\|ROBO1\|CDH8\|PCDH9\|ROBO2\|PVRL3\|PKD1\|CADM4\|PCDH17\|PTPRM\|PCDH7\|FAT4\|IGSF9\|DSG2\|DSC2\|PCDH1\|CLSTN1\|FAT1\|CLSTN2\|PIK3CB\|CLSTN3\|CDHR2 |
|  | GO_DNA_PACKAGING_COMPLEX | 60 | HIST1H2BG\|HIST1H2BJ\|HIST1H2BK\|HIST1H2BI\|HIST1H2BF\|HIST2H2BE\|HIST1H2BC\|NCAPG\|HIST1H4K\|HIST1H3E\|HIST1H2AC\|HIST1H2BO\|H2AFY\|HIST1H3G\|H2AFV\|HIST1H2BL\|HIST1H2AL\|HIST1H4J\|H2AFZ\|H1FX\|HIST4H4\|KAT6A\|HIST1H3D\|H2AFX\|NCAPD2\|NCAPH\|HIST1H3A\|HIST1H3C\|HIST1H1E\|ANKRD32\|KAT6B\|HIST1H3B\|HIST1H2BD\|HIST1H2BH\|SMC4\|HIST1H2AH\|H3F3B\|H2AFY2\|HIST1H4H\|HIST1H4E\|HIST1H4D\|SHPRH\|HIST1H2AG\|SMC2\|HIST1H3H\|H2AFJ\|HIST1H3J\|HIST1H2BN\|HIST3H2BB\|HIST1H1D\|HIST1H1C\|HIST1H1B\|HIST1H3F\|H1F0\|HP1BP3\|MPHOSPH8\|HIST1H4C\|HIST3H2A\|HIST1H2AK\|HIST1H2AE |
|  | GO_TOLL_LIKE_RECEPTOR_SIGNALING_PATHWAY | 57 | PIK3R4\|IRAK1\|IRAK2\|FADD\|IKBKB\|TNIP2\|S100A14\|TRAF6\|HSP90B1\|UBE2D3\|IRAK4\|SCARA3\|BIRC3\|IRF3\|UNC93B1\|COLEC12\|IKBKG\|TBK1\|UBE2D1\|UBE2D2\|CTSB\|TAB2\|TAB3\|RIPK1\|MAP3K1\|CTSK\|UBB\|TICAM1\|CASP8\|RFTN1\|PRKCE\|HSPD1\|CTSS\|BIRC2\|LGMN\|TAB1\|MAPKAPK2\|IKBKE\|UBC\|ITGAM\|RPS27A\|BCL10\|TRAF3\|UBA52\|RPS6KA3\|TANK\|TLR5\|MYD88\|MAP3K7\|CNPY3\|TNIP1\|IRF7\|MAPKAPK3\|TLR2\|RIPK2\|NFKBIA\|PIK3C3 |
|  | GO_POSITIVE_REGULATION_OF_NEURON_DEATH | 54 | ATM\|MAP3K5\|TP53\|CASP3\|CCL3\|APOE\|IKBKB\|PAWR\|TGFB2\|AGER\|CASP9\|TFAP2A\|GSK3B\|EIF2S1\|EPHA7\|ZNF746\|NQO1\|BAX\|ATF2\|ASCL1\|MAP2K7\|FBXW7\|BBC3\|CTNNB1\|DAXX\|ATF4\|TP53BP2\|SRPK2\|FOXO3\|CLU\|DDIT4\|CDK5R1\|ELK1\|CDC42\|CASP2\|PIN1\|AIMP2\|MAP3K11\|CDK5\|UBE2M\|AIFM1\|CDC34\|PICALM\|NF1\|GRIK2\|MCL1\|BCL2L11\|MTOR\|JUN\|DDIT3\|HDAC4\|PARK2\|PMAIP1\|ABL1 |
|  | GO_I_KAPPAB_KINASE_NF_KAPPAB_SIGNALING | 52 | SNIP1\|PTPLAD1\|REL\|NKIRAS1\|IRAK2\|IKBKB\|NFKB1\|TNIP2\|TNF\|TRAF6\|BIRC3\|IKBKG\|TBK1\|DAB2IP\|TAB2\|TAB3\|RIPK1\|UBB\|TICAM1\|BCL3\|BIRC2\|TRAF2\|TAB1\|IKBKE\|RELB\|UBC\|RPS27A\|BCL10\|UBA52\|TANK\|MAP3K7\|RBCK1\|TLR2\|NLRC3\|RIPK2\|ERC1\|PRDX4\|ROCK1\|IRAK1BP1\|NFKB2\|ZNF268\|SHARPIN\|TIFA\|AZI2\|TRIP6\|ZNF675\|TNFRSF1A\|NKIRAS2\|NFKBIL1\|ROCK2\|MAP3K14\|TRADD |
|  | GO_RESPONSE_TO_OSMOTIC_STRESS | 46 | ICOSLG\|SLC12A6\|SERPINB6\|STK39\|MARVELD3\|PCP4\|TRPV4\|TH\|ANXA7\|ITGA2\|MAPK13\|PTK2B\|DDX3X\|SLC2A1\|TSC22D3\|MAP7\|BAX\|RAC1\|SORD\|AKT2\|ATF2\|NFAT5\|MAP2K7\|HSP90AB1\|CCL5\|PKN1\|RELB\|HSP90AA1\|ERRFI1\|FMO1\|TSPO\|HNMT\|TNF\|MYLK\|SLC2A4\|CAB39\|AQP9\|OXSR1\|KMO\|ARHGEF2\|KCNMA1\|LRRC8A\|TSC22D2\|PLK3\|PDPK1\|PKD2 |
|  | GO_REGULATION_OF_NF_KAPPAB_IMPORT_INTO_NUCLEUS | 28 | IL18\|ZC3H12A\|BCL3\|IL1B\|BMP7\|NOL3\|CCDC22\|RBCK1\|SPHK1\|LITAF\|TNF\|CYLD\|TLR2\|IL23A\|EDA\|RHOA\|PPM1B\|NFKBIA\|PRDX1\|ZNF268\|PSMD10\|G3BP2\|FAF1\|TRIP6\|PPM1A\|FBXW11\|NFKBIB\|C8ORF4 |
|  | GO_TUMOR_NECROSIS_FACTOR_RECEPTOR_BINDING | 18 | TRAP1\|TRAF4\|TRIM37\|TRAF1\|ERAP1\|STAT1\|CASP8\|TNF\|TNFSF13B\|TNFSF10\|TRAF6\|EDA\|TRAF2\|TNFSF9\|LTB\|TRAF3\|FADD\|TRADD |
|  | GO_RESPONSE_TO_IMMOBILIZATION_STRESS | 15 | CYP1A1\|TFF1\|GAL\|SLC8A1\|FOS\|TH\|BRD1\|PTK2B\|KEAP1\|PTGER4\|PPP1R15A\|PPARG\|HNMT\|CYP1A2\|LRP11 |

Supplementary material video 1 – Spheroid made of HCT116 cells with red nucleus exposed to NocA at 10µM, for 48h and reinforcement at same concentration for another 48h.

Supplementary material video 2 –Spheroid made of HCT116 cells with red nucleus over 48h with solvent control of NocA and change of medium and respective solvent control concentration of NocA for another 48h.
